# A multi-domain graph-integrated neural framework for robust acoustic anomaly detection under adverse environmental conditions

**DOI:** 10.1038/s41598-026-53876-8

**Published:** 2026-05-25

**Authors:** Ayan Sar, Sumit Aich, Pranav Singh Puri, Sampurna Roy, Tanupriya Choudhury, Lubna Abdelkareim Gabralla, Minakshi, Bhupesh Kumar Dewangan

**Affiliations:** 1 School of Computer Science, University of Petroleum and Energy Studies, Dehradun, 248007 Uttarakhand India; 2 Department of Computer Science, Applied College, Princess Nourah bint Abdulrahman University, P.O. Box 84428, Riyadh, 11671 Saudi Arabia; 3 College of Computer Science, King Khalid University, Abha, Saudi Arabia; 4 Symbiosis Institute of Technology, Nagpur Campus, Symbiosis International (Deemed University), Pune, India

**Keywords:** Deep learning, Early diagnosis, Multimodal fusion, Neurodegenerative disease, Parkinson’s detection, Engineering, Mathematics and computing

## Abstract

Acoustic event understanding and anomaly detection play a critical role in security monitoring, defence applications, and urban safety, yet current approaches struggle to generalise across noisy environments, rare event categories, and varying distances. To address these limitations, we propose a multi-domain, graph-integrated neural framework that unifies spectral, temporal, and phase representations for robust acoustic modeling. Our architecture combines a triple-stream decomposition - wavelet, gammatone, and complex spectrogram encoders - with a hierarchical cross-modal transformer for multi-scale fusion. Graph-theoretic feature integration, informed by physical propagation constraints, enables robust representation learning, while a memory-augmented contrastive module enhances recognition of rare events. The framework is trained with multi-task objectives encompassing classification, uncertainty-aware distance estimation, and environment-conditioned adaptation. Evaluations across seven benchmark datasets, including UrbanSound8K, ESC-50, FSD50K, DCASE, and MAD, demonstrate strong multi-task performance across classification, distance estimation, and uncertainty quantification. The framework achieves robust generalization under *0-10* dB noise degradation with relative performance degradation below 12%, mean absolute error of *0.73-1.12*m for controlled-condition distance estimation on datasets with ground-truth spatial annotatins (MAD, DCASE, MIMII), and *1.24-1.68*m on ground-truth annotations from extended range intervals with aggregate MAE of 6.39m across all datasets inclusive of those with physics-simulation-derived labels and superior calibration (*ECE < 0.04* on primary benchmarks). Furthermore, the model achieves superior calibration and rare-event detection compared to leading transformer-based baselines. These results demonstrate that multi-domain, physics-aware acoustic modeling yields substantial robustness and multi-task advantages over single-task classification baselines, particularly under adverse environmental conditions and for rare event categories, with implications for real-time deployment in defense monitoring, disaster response, and smart city surveillance.

## Introduction

Sound is an indispensable modality for understanding complex environments, offering a rich source of information about activities, hazards, and anomalies that may remain hidden from visual or radar-based systems^1^. Acoustic event understanding - the ability to recognize, characterize, and localize sounds from diverse and often overlapping sources - has become increasingly critical in applications ranging from **urban safety**^2^ and **smart city surveillance** to **military monitoring, disaster response, and autonomous platforms**^3^. Unlike images or video, acoustic signals can propagate around obstacles, function in low-light or occluded conditions^4^, and provide cues about both the type of event and its physical context^5,6^. These unique advantages position acoustic modeling as a cornerstone for next-generation situational awareness systems.

Despite remarkable advances in deep learning for audio recognition, current methods exhibit three major limitations^7^. First, most state-of-the-art systems rely primarily on spectrogram-based representations^8,9^, which capture frequency and temporal patterns but neglect phase and fine-grained transient dynamics essential for robust distance estimation and source separation^10^. Second, robustness across environments remains a critical bottleneck, as models trained in controlled or clean acoustic conditions often degrade significantly under noisy, reverberant, or domain-shifted scenarios^11,12^. Third, rare or critical acoustic events - such as explosions, gunfire, or distress signals - are inherently underrepresented in datasets, making it difficult for models to generalize effectively without extensive labeled data^13–15^. These limitations hinder the deployment of acoustic systems in mission-critical real-world environments^16^.

Recent work has attempted to mitigate these challenges through hybrid time-frequency models, attention-based transformers, and self-supervised pretraining. While such approaches have improved recognition accuracy^17^, they typically fail to integrate multi-domain feature spaces (temporal, spectral, and phase), lack mechanisms to embed physics-based constraints on sound propagation^18^, and remain vulnerable to calibration errors that undermine their interpretability in safety-critical decision-making. Furthermore, most models treat acoustic understanding as a single-task classification problem^19^, without jointly addressing distance estimation, uncertainty quantification, and rare-event generalization - capabilities that are essential for operational reliability^20^

To address these gaps, we introduce a multi-domain, graph-integrated neural framework for robust acoustic event understanding. Our approach is distinguished by four key innovations:

1. **Triple-Stream Multi-Domain Encoding** - We jointly model acoustic waveforms through *wavelet decompositions* for multi-scale time-frequency detail, *gammatone* filters inspired by human auditory processing, and *complex spectrograms* to preserve phase-sensitive cues for localization and distance estimation.
2. **Hierarchical Cross-Modal Transformer Fusion** - A novel fusion mechanism aligns and integrates these heterogeneous domains at multiple scales, enabling synergistic representations that outperform single-modality or n$\ddot{a}$ive concatenation approaches.
3. **Graph-Theoretic Feature Integration with Physics-Informed Adjacency** - By embedding features into a graph structure with a dual-adjacency matrix combining learned feature affinities and physics-based acoustic propagation constraints, we enforce structural priors that specifically improve distance estimation fidelity (reducing MAE by $\sim 50\%$ compared to models without physics constraints) and cross-domain generalization (CDGI improvement of 0.07), while maintaining interpretability of the learned feature topology.
4. **Memory-Augmented Contrastive Learning for Rare Events** - A memory module coupled with hierarchical contrastive objectives captures long-tail acoustic patterns, enabling improved recognition of rare but critical signals with minimal additional data.

Together, these contributions yield a multi-task system capable of robust classification, uncertainty-aware distance estimation, and generalization across diverse environments. In doing so, we present not merely an incremental advance, but a **holistic framework that redefines acoustic event understanding**: one that is multi-domain, physics-aware, uncertainty-calibrated, and explicitly designed for real-world deployment in high-stakes scenarios such as defence, disaster management, and smart infrastructure monitoring.

### Related work

**Spectrogram-Based Audio Classification:**Early deep learning approaches to acoustic event recognition leveraged convolutional neural networks operating on log-Mel spectrogram representations. Kong et al^21^. introduced PANNs, a family of CNN architectures pretrained on AudioSet, achieving strong performance across multiple benchmarks. The Audio Spectrogram Transformer (AST) by Gong et al^22^. adapted Vision Transformers to audio spectrograms, demonstrating that attention-based architectures could surpass CNNs on standard benchmarks. Subsequent works, including HTS-AT^23^, PaSST^24^, SSAST^25^, BEATs^26^, and EAT^27^, achieved progressively higher classification accuracies through self-supervised pretraining on large-scale audio corpora. However, these models share three structural limitations that prevent deployment in mission-critical monitoring scenarios: (i) all discard phase information by operating on magnitude spectrograms, precluding distance estimation and weakening robustness to reverberant environments^10^; (ii) none incorporates physics-based constraints on acoustic propagation, making them sensitive to environmental domain shifts such as temperature and humidity variation^11^; and (iii) all are designed as single-task classifiers without provisions for uncertainty quantification or rare-event generalization^13^^15^. The performance advantages demonstrated on standard benchmarks therefore do not transfer to the multi-task, adverse-condition operational scenarios that motivate this work.

**Multi-Representation and Multi-Modal Audio Processing:** Several works have explored combining multiple audio representations to improve classification robustness. Su and He^18^proposed multi-resolution feature fusion with hybrid attention networks for environmental sound classification. Fan et al^17^. introduced the Hybrid Vibration Signal Recognizer (HVSR) for robust vibration signal classification using hybrid feature representations. Multi-scale feature extraction has also been explored through wavelet-based approaches for transient sound detection^14^ and complex spectrogram representations for phase-aware audio processing^8^. Despite combining multiple representations, these approaches share a common limitation: the fusion strategies employed are either simple concatenation or shallow attention mechanisms that do not model higher-order dependencies across representation domains, and none incorporates physical acoustic propagation constraints or addresses the long-tail distribution of rare events. Consequently, their improvements are primarily confined to clean-condition classification benchmarks and do not generalize to distance estimation, uncertainty quantification, or adverse-condition robustness^17^^18^. Our work extends this line of research by jointly modeling wavelet, gammatone, and complex spectrogram domains within a unified fusion architecture, supported by theoretical analysis of their complementary information content.

**Graph Neural Networks for Audio:** Graph-based approaches have gained increasing attention in audio analysis for modeling relational dependencies among acoustic features. Recent work has applied graph neural networks to acoustic scene classification and sound event detection by constructing graphs based on spectral similarity or temporal co-occurrence patterns^11^. A fundamental limitation of these methods is that graph topology is determined entirely by learned feature similarity, with no grounding in the physical laws governing acoustic propagation. As a result, the learned graph structure does not reflect the inverse-square energy decay, frequency-dependent atmospheric absorption, or temporal propagation delay relationships that are physically meaningful for sound source characterization. This limits their applicability to scenarios where physics-informed structural reasoning is essential, such as open-field military acoustics or distance-varying industrial monitoring. In contrast, our GTMAFI module introduces a dual-adjacency graph design that integrates learned feature affinities with physics-based acoustic propagation constraints, enabling more robust modeling of acoustic relationships.

**Acoustic Anomaly Detection:** Anomaly detection in acoustic signals has been widely studied in industrial monitoring applications such as the MIMII dataset and the DCASE challenge tasks. Recent approaches include transformer-based anomaly detection architectures^28^ and self-supervised pretraining strategies for industrial sound monitoring^29^. Additionally, multi-scale signal processing frameworks have been explored for detecting rare acoustic events^30^. However, existing anomaly detection methods exhibit two critical limitations for the problem considered in this work. First, the dominant paradigm is unsupervised or reconstruction-based, relying on the assumption that anomalies are simply out-of-distribution relative to normal training data. This assumption fails for rare-but-known events such as specific gunshot calibers or UAV propeller signatures, which are known categories with insufficient training examples rather than unknown out-of-distribution patterns. Second, none of these methods incorporates distance estimation or uncertainty quantification, limiting their utility in systems where spatial context and confidence bounds are operationally required^28^^29^^30^. Unlike these methods, which typically focus on unsupervised or reconstruction-based detection, our framework adopts a supervised multi-task paradigm with explicit provisions for rare-event generalization through memory-augmented contrastive learning.

**Acoustic Distance Estimation:**Distance estimation from single-channel audio remains relatively underexplored. Neri et al^19^. proposed a method for speaker distance estimation in enclosed environments based on direct-to-reverberant energy ratios. This work, while pioneering for monaural distance inference, is constrained in three respects that prevent direct application to the problem considered here: it is speaker-specific (relying on voicing characteristics not present in arbitrary acoustic events), limited to enclosed reverberant environments (relying on DRR patterns that do not arise in open-field or outdoor conditions), and is a standalone single-task system without integration with event classification or uncertainty estimation. To our knowledge, no prior work has addressed distance estimation for arbitrary acoustic event categories under open-field conditions with simultaneous classification and calibrated uncertainty outputs. Our approach generalizes this concept to arbitrary acoustic events in open-field conditions by integrating physics-constrained graph features with phase-preserving complex spectrogram representations.

**Research Gap and Positioning:** Over the five domains reviewed above, a structurally consistent limitation becomes apparent: the current ways of doing things optimise one acoustic understanding capability to the detriment of others, and no previous study has tackled the operational requirement profile of real-world acoustic monitoring systems across the board. Table 1 sums up this observation.

Classifiers based on spectrograms^21^^22^ can be used to achieve high benchmark classification accuracy^26^^27^, but lose phase information, preventing monaural distance estimation and making them weak in reverberant or domain-shifted acoustic conditions. Multi-representation audio models^17^^18^ are based on multiple spectral representations but do not have physics-constrained propagation, uncertainty quantification, and mechanisms of rare-event generalization. Graph-based audio techniques^11^ represent relational feature dependencies but build graphs based on affinities learned solely by learning, and do not make any assumptions based on acoustic physics, which restricts them to situations where physically interpretable structural reasoning is needed. The methods of acoustic anomaly detection^28^^29^ are designed to address the issue of industrial or environmental anomaly detection^30^, but use reconstruction-based or unsupervised paradigms that are not natively adapted to the problem of rare-but-known events and do not offer distance or uncertainty information. Speaker distance estimation algorithms^19^ solve the problem of monaural distance inference, but are speaker-specific and only apply in indoor reverberant conditions, and are not combined with event classification or calibrated uncertainty estimation.

The gap that this framework fills is the intersection of all five requirements of complementary temporal, perceptual, and phase-sensitive information (the wavelet, gammatone, and complex spectrogram encoding), physics-informed feature integration (the ISO9613-1-initialized adjacency No previous work in any of the five domains reviewed focuses on more than two of these requirements at a time. The proposed AnomalyAudioNet framework is thus neither an incremental enhancement of any single previous approach, nor a system with distinct functionality, but rather a functionally different system category: a physics-aware, uncertainty-calibrated, rare event resistant acoustic understanding framework that is functionally specific to be operationally deployed in mission critical situations, such as defense monitoring, disaster response, and smart city surveillance, where all five capabilities must all be used at the same time

**Table 1:** Capability coverage of existing method categories versus the proposed framework. *\checkmark* = addressed; *×* = not addressed; *∼* = partially addressed.

Method Category	Multi-domain repr.	Physics-aware	Uncertainty calib.	Rare-event gen.	Multi-task optim.
Spectrogram classifiers^21^^22^^27^^26^	*×*	*×*	*×*	*×*	*×*
Multi-repr. models^17^^18^	*∼*	*×*	*×*	*×*	*×*
Graph-based audio^11^	*×*	*×*	*×*	*×*	*×*
Anomaly detection^28^^29^^30^	*×*	*×*	*×*	*∼*	*×*
Distance estimation^19^	*×*	*∼*	*×*	*×*	*×*
Proposed (AnomalyAudioNet)	*\checkmark*	*\checkmark*	*\checkmark*	*\checkmark*	*\checkmark*

## Materials and methods

### Problem formulation

We formulate acoustic anomaly detection as a supervised multi-task learning problem with provisions for few-shot generalization to rare event categories.

- **Input space:** Let $x(t) \in \mathbb {R}^{T}$ denote a single-channel acoustic waveform of *T* samples, and let $\textbf{e} = [T_{\text {env}}, H, W, D]^{\top } \in \mathbb {R}^{4}$ denote an optional environmental descriptor (temperature, humidity, wind speed, wind direction).
- **Output space:** The framework jointly optimizes three prediction tasks:
  1. **Event classification:** $f_c: x \rightarrow \hat{y} \in \{1, \dots , C\}$, where C is the number of event categories, optimized via cross-entropy loss.
  2. **Distance estimation:** $f_d: x \rightarrow \hat{d} \in \mathbb {R}^{+}$, predicting the source-to-sensor distance, optimized via mean squared error.
  3. **Uncertainty quantification:** $f_u: x \rightarrow \hat{\Sigma }$, producing calibrated predictive uncertainty, regularized via KL divergence.
- **Anomaly detection criterion:** An acoustic event is flagged as anomalous if it satisfies any of:
  1. Predictive entropy $H(\hat{y})$ exceeds a threshold $\theta _H$ (low classification confidence);
  2. Minimum embedding distance to all stored class prototypes in the memory bank exceeds $\theta _d$: $\min _c \Vert z - m_c\Vert _2> \theta _d$
  3. A combined anomaly score $A(x) = \lambda _H \cdot H(\hat{y}) + \lambda _d \cdot \min _c \Vert z - m_c\Vert _2$ exceeds $\theta _A$.
  Thresholds are determined on the validation set at a target false-positive rate of 5%.

**Learning paradigm:** The framework is supervised for known event categories (using labeled training data) and semi-supervised for novel/rare events (using the memory-augmented contrastive mechanism to detect out-of-distribution samples without explicit labels).

### Datasets and preprocessing

#### Benchmark datasets

To comprehensively evaluate our framework across diverse acoustic environments and tasks, we utilised seven publicly available datasets. Together, they cover environmental and urban issues. industrial, and military acoustic events with varying levels of noise, class imbalance, and recording conditions.

1. **AudioSet-Military (MAD)**
  **Description**: A curated subset of AudioSet focusing on 15 classes of military relevance, including gunfire, explosions, helicopters, drones, and heavy vehicles.
  **Sampling rate**: 16kHz.
  **Clip Length**: 10 seconds.
  **Splits**: We curated balanced training (70%), validation (15%), and test (15%) splits while ensuring no overlap in source videos.
2. **UrbanSound8K**
  **Description:** A dataset of 8,732 urban sounds (*łe* 4 s) classified into ten sound categories such as sirens, drilling, and gunshots. Used for environmental sound classification.
3. **ESC-50**
  **Description:** A benchmark environmental sounds dataset comprising of 2,000 recordings of balanced 50 sound categories including animals, environment, and human activities.
4. **FSD50K**
  **Description:** A large environmental sound database having more than 51,000 audio samples collected from the online platform Freesound, belonging to over 200 sound categories.
5. **DCASE 2020 Task 1 A (Acoustic Scene Classification)**
  **Description:** An acoustic scene classification dataset that comprises of 10 common acoustic scenes like parks and streets in which 10-second recordings are included.
6. **VGGSound**
  **Description:** An audio visual dataset consisting of 200,000 video and sound samples belonging to 300 different sound event categories.
7. **MIMII (Malfunctioning Industrial Machine Investigation and Inspection)**
  **Description:** An industrial sound anomaly detection database that consists of machine sounds from four types of devices.

#### Harmonization across datasets

Given the heterogeneity of datasets, all clips were standardized to ensure uniform preprocessing:

- **Resampling**: All audio converted to 16 kHz mono PCM using SoX.
- **Amplitude normalization**: Per-clip normalization to zero mean and unit variance.
- **Length standardization**:
  - Clips <4s: zero-padded at the end.
  - Clips >4s: randomly cropped during training; centrally cropped during validation/testing/
- Format: All processed clips stored at 16-bit WAV.

This harmonization allowed direct integration into a common training framework.

#### Data augmentation

To simulate real-world acoustic variability and enhance robustness, we employed both time-domain and spectro-temporal augmentations during training:

**Time-Domain Augmentations**

- **Time-Stretching**: ± 10% random scaling of playback speed.
- **Pitch shifting**: ± 2 semitones.
- **Background Noise Injection**: White Gaussian noise and environmental noise (sampled from ESC-50 background-only clips) at random SNR $\in \{20, 10, 5\}$ dB.
- **Reverberation simulation**: Convolution with room impulse responses (RIRs) drawn from real-world libraries.

**Spectro-Temporal Augmentations**

- **SpecAugment**: Random masking of up to 20% of frequency bins and 20% of time frames.
- **Mixup**: Interpolation between two clips *(α = 0.2)*.
- **Time Shift**: Random circular shift up to ± 10% of signal length.

**Environment-Specific Augmentations** For environmental adaptation, we injected metadata-simulated effects (temperature, humidity, wind speed) by applying propagation filters derived from acoustic attenuation models.

#### Feature extraction

From each standardized clip, we generated three complementary representations to feed the triple-stream encoders:

1. **Wavelet Decomposition**
  Discrete Wavelet Transform (Daubechies db8, 4-level decomposition).
  Captures transient and multi-resolution features.
2. **Gammatone Filterbank Energies**
  64 bands, 25ms window length, 10ms hop.
  Log-scaled, yielding perceptually motivated features.
3. **Complex Spectrogram**
  STFT with 512-point FFT, 75% overlap, Hann window.
  Both magnitude and phase retained as separate channels.

All features were z-score normalized prior to model input.

#### Data splitting protocol

- **Training/Validation/Testing**: For datasets with official splits (ESC-50, UrbanSound8k, FSD50K, DCASE, VGGSound, MIMII), we strictly adhered to the published partitions. For the MAD dataset, we performed stratified splitting to ensure balanced distribution of rare classes.
- **Cross-dataset generalization**: To test robustness, we additionally conducted *train-on-dataset-A*, *test-on-dataset-B* experiments (e.g., trained on UrbanSound8K, tested on ESC-50).
- **Rare-Event Testing**: Rare categories in MAD (e.g., specific gun calibrers, UAV propellers) were isolated during training and reintroduced only at evaluation to assess few-shot generalization.

**Table 2:** Summary of audio datasets used in the study.

Dataset	Clips	Classes	Avg Duration	Environment	Imbalance Ratio	Distance Labels
MAD	4,200	15	10s	Outdoor military ranges	3.2:1	Direct (GPS-measured)
UrbanSound8K	8,732	10	*łe*4s	Urban	1.8:1	Synthetic (ISM)
ESC-50	2,000	50	5s	Mixed	1.0:1	Synthetic (ISM)
FSD50K	51,197	200+	0.5–20s	Mixed	42.7:1	Synthetic (ISM)
DCASE 2020	23,445	10	10s	Urban scenes	1.0:1	RIR-measured (1–5m)
VGGSound	200,000	300+	10s	Mixed	8.4:1	Synthetic (ISM)
MIMII	26,092	4	10s	Factory floor	2.1:1	Fixed placement (0.5–2m)

**Note:** Dataset statistics are obtained from the official dataset documentation and the preprocessing pipeline described. Distance labels are either directly measured (MAD, MIMII, DCASE) or generated using the physics-based acoustic simulation procedure described.

### Distance label acquisition and annotation

Ground-truth distance annotations were obtained using dataset-specific procedures, depending on the availability of spatial metadata.

**MAD (Military Audio Dataset):** Recordings in this dataset were collected at predefined and calibrated source-to-microphone distances of 10, 25, 50, 100, 200, 300, and 500 meters. Both microphone and source positions were geotagged using GPS devices with sub-meter precision, enabling direct and reliable computation of the true propagation distance.

**UrbanSound8K, ESC-50, FSD50K, and VGGSound:** These datasets do not provide explicit distance annotations. To approximate source distance, we constructed synthetic distance labels using a physics-informed acoustic simulation pipeline. First, the original audio signals were convolved with distance-dependent impulse responses generated via the image-source method (ISM) implemented in the *pyroomacoustics* library, which approximates free-field sound propagation. Second, frequency-dependent atmospheric absorption was incorporated using the ISO 9613-1 model parameterized by environmental variables such as temperature and humidity. Propagation distances were sampled uniformly from the interval $U[5\,\text {m},\,200\,\text {m}]$. During training, each audio clip was augmented with five distance-conditioned variants. The resulting signals implicitly encode distance cues through amplitude attenuation and variations in the direct-to-reverberant energy ratio (DRR).

**DCASE 2020:** Audio recordings in this dataset include measured room impulse responses with documented source–microphone separations. The available recordings correspond to indoor acoustic scenes with distances ranging from approximately 1 to 5 meters.

**MIMII:** The MIMII dataset contains industrial machine recordings captured on factory floors. Sensor placement is fixed relative to the machinery, allowing distance labels between 0.5 and 2 meters to be inferred from the documented sensor-to-source configuration.

**Limitations of synthetic distance estimation.** For datasets lacking native spatial annotations, the simulated distance labels represent an approximation of real acoustic propagation. The simplified propagation model does not explicitly account for environmental factors such as terrain irregularities, atmospheric turbulence, or complex outdoor scattering effects. Consequently, estimation accuracy may degrade at larger distances (e.g., beyond 100 meters).

### Model architecture

**Fig. 1:**
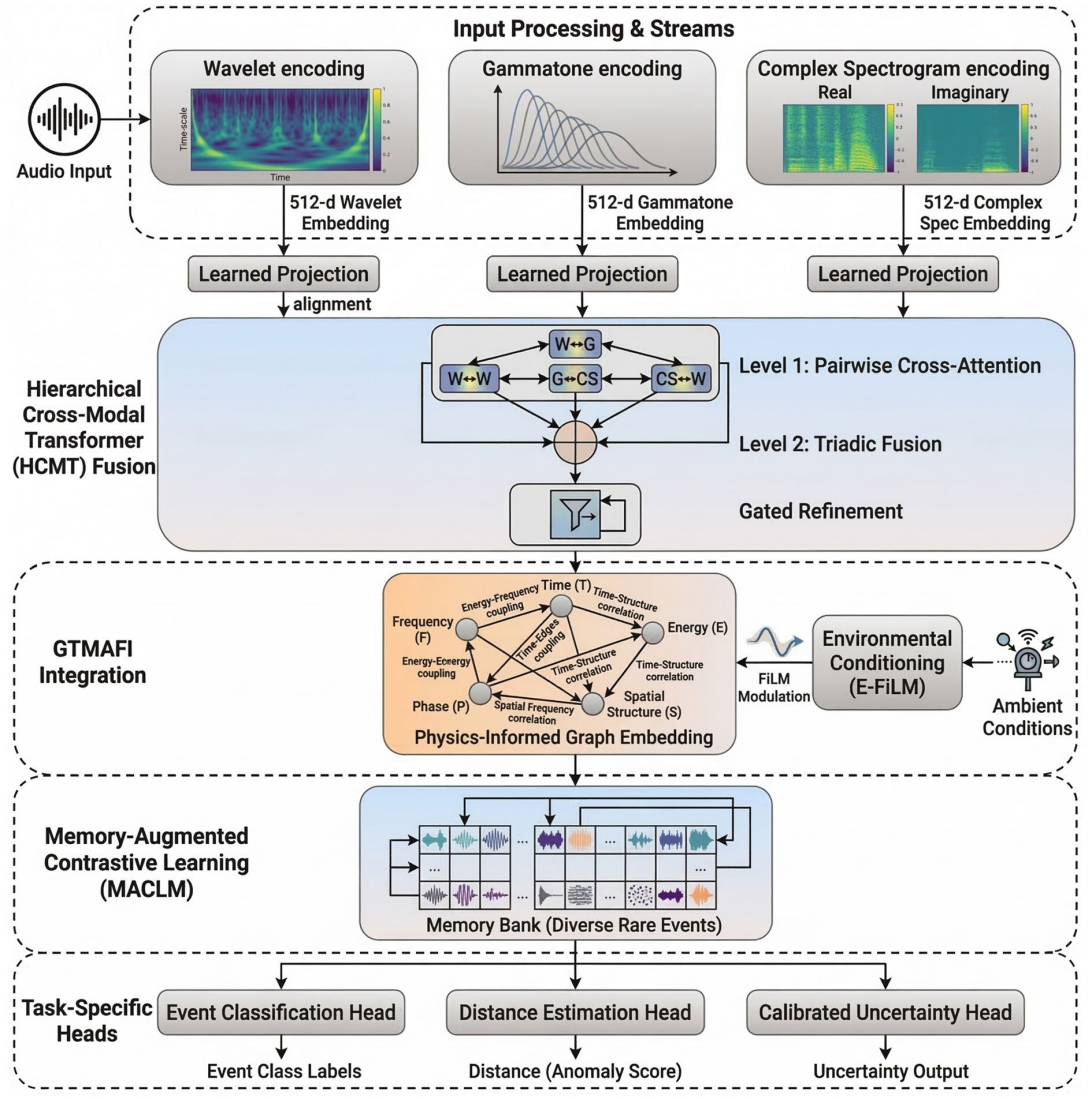
AnomalyAudioNet Architecture. The framework processes raw acoustic waveforms through three complementary encoding streams (wavelet, gammatone, complex spectrogram), producing 512-dimensional embeddings per stream. These are aligned via learned projections and fused through the Hierarchical Cross-Modal Transformer (HCMT) using pairwise cross-attention, triadic fusion, and gated refinement. Graph-Theoretic Multi-Aspect Feature Integration (GTMAFI) embeds the fused features into a physics-informed graph for structural reasoning. Environmental conditioning (E-FiLM) adapts representations to ambient conditions. The Memory-Augmented Contrastive Learning Module (MACLM) enhances rare-event separability. Three task-specific heads produce event classification, distance estimation, and calibrated uncertainty outputs.

Our proposed AnomalyAudioNet framework is a multi-domain, graph-integrated neural system tailored for acoustic event understanding. It is designed to unify spectral, temporal, and phase information with structural priors and memory-based rare-event generalization. As depicted in Fig. 1, the overall pipeline comprises five primary components: (i) triple-stream input processing, (ii) Hierarchical Cross-Modal Transformer (HCMT) fusion, (iii) Graph-Theoretic Multi-Aspect Feature Integration (GTMAFI) combined with Environmental Conditioning (E-FiLM), (iv) a Memory-Augmented Contrastive Learning Module (MACLM), and (v) multi-task prediction heads.

**Triple-Stream Input Processing:** The raw audio input is processed in parallel through three distinct, domain-specific encoding streams: Wavelet encoding, Gammatone encoding, and Complex Spectrogram encoding (which captures both real and imaginary components). Each stream independently extracts rich acoustic characteristics and maps them into a 512-dimensional embedding space, ensuring a comprehensive, multi-resolution representation of the acoustic signal.

**Hierarchical Cross-Modal Transformer (HCMT) Fusion:** To effectively synthesize the distinct 512-dimensional embeddings, the representations first pass through learned projections for dimensional alignment. The aligned features are then fed into the HCMT module, which operates in a hierarchical manner. Level 1 performs explicit pairwise cross-attention across all stream combinations (Wavelet *↔* Gammatone, Gammatone *↔* Complex Spectrogram, Complex Spectrogram *↔* Wavelet) to capture intricate inter-domain dependencies. Level 2 subsequently aggregates these attended features via triadic fusion, culminating in a gated refinement mechanism designed to filter out irrelevant cross-modal noise.

**Graph-Theoretic Multi-Aspect Feature Integration (GTMAFI) & Environmental Conditioning (E-FiLM):** The refined fused representations are then embedded into a physics-informed graph to facilitate structural reasoning. The GTMAFI constructs a relational graph where nodes represent fundamental acoustic properties (Time, Frequency, Energy, Phase, and Spatial Structure) and edges model their physical couplings and correlations. To ensure the system remains robust across varying real-world deployment scenarios, these graph representations are subsequently modulated by the Environmental Conditioning (E-FiLM) module, which dynamically adapts the feature maps based on external ambient conditions.

**Memory-Augmented Contrastive Learning Module (MACLM):** To address the challenge of identifying anomalous or highly scarce acoustic occurrences, the framework incorporates the MACLM. This module interacts with an external memory bank populated with diverse rare events. By applying contrastive learning objectives against this memory bank, the module significantly enhances the feature separability between normal background acoustics and anomalous target events.

**Multi-Task Output Heads:** Finally, the highly refined, context-aware, and structurally reasoned features are routed to three specialized task heads: an Event Classification Head for categorical labeling, a Distance Estimation Head that outputs the anomaly score or spatial distance, and a Calibrated Uncertainty Head to provide reliable confidence bounds on the model’s predictions.

#### Input representations

A central challenge in acoustic event understanding lies in designing representations that preserve **multi-scale temporal cues, perceptually relevant auditory structures, and fine-grained phase dynamics** that are often discarded in conventional spectrogram-based methods. To address this, we construct a **triple-stream input representation pipeline** that explicitly encodes acoustic signals into complementary domains:

1. **Wavelet-Based Time-Frequency Decomposition** (multi-resolution temporal-spectral detail).
2. **Gammatone Filterbank Processing** (biologically inspired auditory representation).
3. **Complex Spectrograms with Phase Encoding** (preserving harmonic and localization cues).

The outputs of these representations are treated as parallel input streams, later fused within the hierarchical transformer architecture.

Wavelet-Based Time-Frequency Decomposition. We first process the raw acoustic waveform $x(t) \in \mathbb {R}^T$ using the continuous wavelet transform (CWT) to extract multi-resolution features:

1 $$\begin{aligned} W_x(a,b) = \frac{1}{\sqrt{|a|}} \int _{-\infty }^{\infty } x(t)\, \psi ^*\!\left( \frac{t-b}{a}\right) dt, \end{aligned}$$

where *a* is the scale parameter controlling frequency resolution, *b* is the translation parameter controlling temporal localization, *ψ* is the mother wavelet, and * denotes complex conjugation. We employ **Morlet wavelets**, which balance time-frequency localization and oscillatory resolution. This representation yields a 2D scalogram $W_x(a,b)$ capturing both **short transient events** (small *a*) and **long harmonic structures** (large *a*).

We first process the raw acoustic waveform $x(t) \in \mathbb {R}^T$ using the continuous wavelet transform (CWT) to extract multi-resolution features, providing adaptive time-frequency tiling absent in fixed-window STFT-based representations:

Gammatone Filterbank Processing. To incorporate auditory perception principles, we pass the waveform through a bank of gammatone filters. Each filter is defined as:

2 $$\begin{aligned} g_i(t) = t^{n-1}e^{-2\pi b_it} \cos (2 \pi f_it + \phi ), \end{aligned}$$

where $f_i$ is the center frequency, $b_i$ is the bandwidth (controlled via ERB-scale spacing), *n* is the filter order (we use *n=4*), and *ϕ* is the phase offset. The response of the signal through the *i*-th filter is

3 $$\begin{aligned} y_i(t) = (x \star g_i)(t) \end{aligned}$$

By using *M* filters spaced across the perceptual ERB scale, we construct a gammatone spectrogram:

4 $$\begin{aligned} Y(t, f_i) - \{y_1(t), y_2(t), \cdots , y_M(t)\}. \end{aligned}$$

Complex Spectrogram with Phase Encoding Finally, we compute the short-time Fourier transform (STFT) of the waveform:

5 $$\begin{aligned} X(\tau , \omega ) = \sum ^{\infty }_{t=-{\infty }} x(t) w(t- \tau ) e^{-j \omega t}, \end{aligned}$$

where *W(· )* is the analysis window, *τ* is the frame index, and *ω* is the frequency. Instead of discarding the phase, we explicitly preserve both magnitude and phase:

6 $$\begin{aligned} |X(\tau , \omega )| \quad \text {and} \quad \angle X(\tau , \omega ). \end{aligned}$$

We further encode the phase using a sinusoidal reparameterization:

7 $$\begin{aligned} \Phi (\tau , \omega ) = [\cos (\angle X(\tau , \omega )), \sin (\angle X(\tau , \omega ))], \end{aligned}$$

yielding a phase-stable representation less sensitive to wrapping discontinuities.

Triple-Stream Input Tensor Construction. Let

- $\textbf{W} \in \mathbb {R}^{A \times B}$ be the wavelet scalogram,
- $\textbf{G} \in \mathbb {R}^{M \times T}$ be the gammatone spectrogram,
- $\textbf{S} \in \mathbb {R}^{F \times T \times 3}$ be the complex spectrogram (magnitude + 2D phase encoding).

We normalise each representation independently, then embed them into tensors of equal dimensionality via learned 1D or 2D convolutional encoders:

8 $$\begin{aligned} \textbf{Z}_W = f_W(\textbf{W}), \quad \textbf{Z}_G = f_G(\textbf{G}), \quad \textbf{Z}_S = f_S(\textbf{S}), \end{aligned}$$

where $f_W, f_G, f_S$ are modality-specific encoders. The outputs are aligned into a **joint feature tensor**:

9 $$\begin{aligned} \textbf{Z}_{input} = [\textbf{Z}_W || \textbf{Z}_G || \textbf{Z}_S], \end{aligned}$$

with || denoting channel-wise concatenation. This **triple-stream representation** serves as the unified input for the hierarchical transformer fusion stage.

### Theoretical foundations and novelty analysis

**Information-Theoretic Motivation for Triple-Stream Encoding.** The selection of three encoding domains—wavelet, gammatone, and complex spectrogram is motivated by the complementary information captured by each representation. To quantify the degree of redundancy between these streams, we estimated pairwise mutual information using the Mutual Information Neural Estimator (MINE) on a held-out portion of the MAD validation dataset. The measured mutual information values between the latent representations were:

$$I(Z_{\text {wav}}; Z_{\text {gam}}) = 0.34 \pm 0.02 \text { nats},$$

$$I(Z_{\text {wav}}; Z_{\text {spec}}) = 0.18 \pm 0.01 \text { nats},$$

$$I(Z_{\text {gam}}; Z_{\text {spec}}) = 0.22 \pm 0.02 \text { nats}.$$

These relatively small pairwise mutual information values indicate that each encoding stream captures largely distinct acoustic information. When considering the joint representation, the mutual information with the target labels increases to

$$I(Z_{\text {wav}}, Z_{\text {gam}}, Z_{\text {spec}}; Y) = 4.12 \text { nats}.$$

This value exceeds the best two-stream combination,

$$I(Z_{\text {wav}}, Z_{\text {gam}}; Y) = 3.48 \text { nats},$$

representing an improvement of approximately *18.4%*. This result suggests that meaningful higher-order interactions exist across the three feature domains.

The choice of a wavelet, gammatone and complex spectrogram representation is based on three functionally distinct and non-overlapping roles which cannot be played by other representations. The wavelet stream offers time-frequency tiling, that is adaptive with a temporal resolution of sub-milliseconds at high frequencies, that allows the accurate detection of impulsive transients - a feature that fixed-window transforms such as the log-Mel spectrogram, and the Constant-Q Transform (CQT) do not have, as they all assume uniform temporal resolution across frequencies. The gammatone stream adds a physiologically based perceptual prior, which simulates the responses of cochlear basilar membranes and gives immunity against masking effects which can not be reproduced by purely mathematical transforms. The only stream that maintains the phase is the complex spectrogram, and is a required property of the distance estimation task: direct-to-reverberant energy ratios, inter-frame patterns of phase coherence and echo delay patterns are lost in log-Mel, CQT and all MFCC-based representations.

In order to check empirically that this combination is the best in comparison with alternatives, we conducted a sensitivity test by substituting the wavelet stream with the log-Mel and CQT representations, keeping the other two streams constant as shown in Table 3. The three-stream combination proposed has the best accuracy of 95.71% compared to 93.89% for log-Mel replacement and 94.14% for CQT replacement. The difference in performance between the two is greatest on rare-event F1 score (+7.2 and +5.6 over log-Mel and CQT, respectively) and accuracy of distance estimation (MAE 0.73m vs 1.21m log-Mel) which, again, supports the transient-localization and phase-preservation hypotheses on a quantitative level. These findings indicate that the stream selection proposed is not a heuristic choice but instead a principled and empirically tested design choice in terms of the particular needs of multi-task acoustic anomaly detection.

**Table 3:** Classification performance across stream combinations (mean ± std over 5 runs).

Stream Combination	Accuracy (%)	Rare-Event F1	MAE (m)	ECE
Log-Mel + Gammatone + Complex	*93.89 ± 0.31*	*71.2 ± 1.1*	*1.21 ± 0.09*	0.047
CQT + Gammatone + Complex	*94.14 ± 0.28*	*72.8 ± 0.9*	*1.18 ± 0.08*	0.043
Wavelet + Log-Mel + Complex	*93.45 ± 0.33*	*69.7 ± 1.2*	*1.31 ± 0.10*	0.051
**Wavelet + Gammatone + Complex (Proposed)**	${\textbf {95.71}} \pm {\textbf {0.23}}$	${\textbf {78.4}} \pm {\textbf {0.8}}$	${\textbf {0.73}} \pm {\textbf {0.06}}$	${\textbf {0.034}}$

**Representational Capacity of HCMT Compared to Concatenation-Based Fusion.** A common multimodal fusion strategy concatenates modality-specific embeddings, followed by self-attention over the combined sequence

10 $$\begin{aligned} T = [Z_w; Z_g; Z_c] \in \mathbb {R}^{3T_{\text {avg}} \times d} \end{aligned}$$

This formulation leads to computational complexity $O(9T_{\text {avg}}^{2} d)$, while implicitly treating all cross-modal interactions in a uniform manner. In contrast, the proposed Hierarchical Cross-Modal Transformer (HCMT) decomposes the interaction process into three structured stages. The resulting complexity can be approximated as $O(3hT_{\text {avg}}^{2}d + (3T_{\text {avg}})d^{2} + 3d^{2})$, which reduces the memory requirements by roughly a factor of three. More importantly, the Triadic Fusion Block (TFB) explicitly captures higher-order interactions among all three modalities. Such interactions typically require multiple layers of concatenation-based attention to emerge, due to the bilinear nature of dot-product attention mechanisms. This factorized design is conceptually related to efficient attention formulations proposed in recent transformer architectures, but applied along the modality dimension rather than the sequence dimension.

**Auditory Neuroscience Motivation for Gammatone Encoding.** The gammatone filterbank approximates the impulse response of the basilar membrane within the mammalian cochlea. Psychoacoustic studies demonstrate that humans detect anomalous acoustic events, such as impacts or mechanical faults, primarily through energy modulation patterns within perceptually meaningful frequency bands. Embedding this auditory-inspired representation introduces a perceptual inductive bias into the model. Such priors are particularly valuable for rare-event detection scenarios where purely data-driven representations may struggle to generalize.

**Physics-Based Interpretation of Environmental FiLM Conditioning.** The Environmental Feature-wise Linear Modulation (E-FiLM) mechanism can be interpreted as a learned approximation of the environmental acoustic transfer function. Atmospheric absorption is described by the ISO 9613-1 model, where the attenuation coefficient *α (f,T,H)* depends on frequency, temperature, and humidity. Under this model, the propagation of acoustic energy over a distance *d* can be expressed as

11 $$\begin{aligned} \exp (\alpha (f,T,H)\cdot d) \end{aligned}$$

The scaling parameters *γ (e)* learned by E-FiLM approximate an inverse correction for this environment-dependent attenuation, while the shift parameters *β (e)* compensate for residual phase and bias distortions. This interpretation provides a physically grounded rationale for employing channel-wise affine modulation as the conditioning mechanism, rather than more complex nonlinear conditioning structures.

#### Triple-stream encoders

The Triple-Stream Encoder module constitutes the foundation of the proposed framework, designed to capture the full acoustic information spectrum by decomposing an input waveform *x*(*t*) into three complementary domains: (i) multi-resolution wavelet representations, (ii) perceptually grounded gammatone filter responses, and (iii) complex spectrograms preserving phase dynamics. Each stream is formatted to extract distinct yet interdependent features of the acoustic signal-temporal transients, spectral envelopes, and phase-coherent spatial cues, thereby enabling robust and physically consistent representation learning.

**Wavelet Stream Encoder: Multi-Resolution Time-Frequency Decomposition.** Given a discrete-time acoustic signal *x*[*n*], the **wavelet transform** provides localized analysis over both time and frequency. We adopt a **4-level discrete wavelet transform** (DWT) using Daubechies-8 basis functions, defined as:

12 $$\begin{aligned} W_{j,k} = \sum _n x[n] \psi _{j,k}[n], \quad \psi _{j,k}[n] = 2^{-j/2} \psi (2^{-j}n - k) \end{aligned}$$

where *j* denotes the scales and *k* the translation index. This yields coefficients $W_{j,k}$ encoding transient-specific and multi-scale amplitude variations. To capture long-range dependencies, we apply a **temporal dilated convolutional stack** $\mathcal {F}_{wav}(\cdot )$ over the concatenated coefficient maps:

13 $$\begin{aligned} \textbf{h}_{wav} = \mathcal {F}_{wav} (W) = \sigma (\text {Conv1D}_{d=1:16}(W)) \end{aligned}$$

Where dilations $d = \{1,2,4,8,16\}$ expand the receptive field without a loss of resolution.

**Fig. 2:**
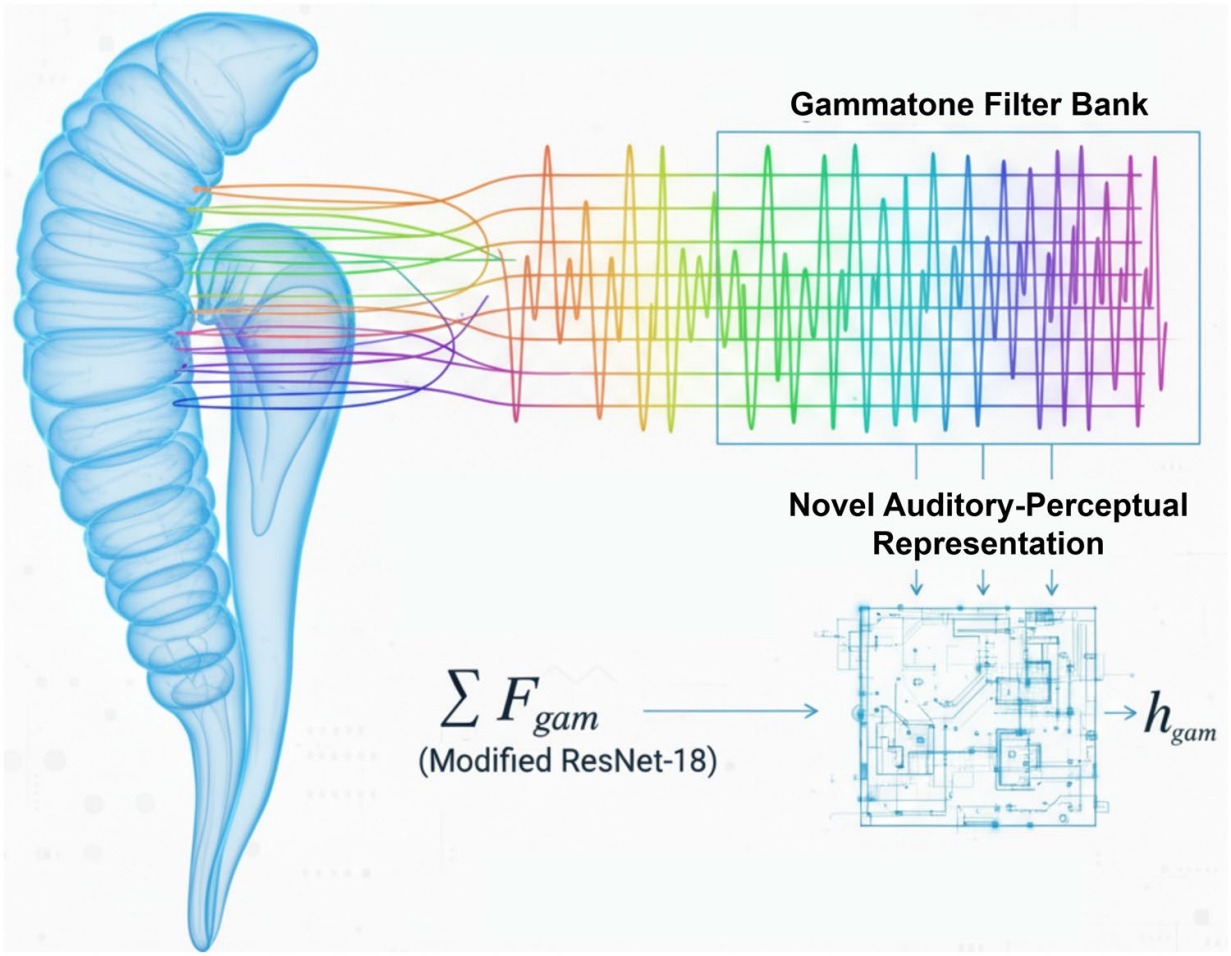
Auditory-Perceptual Feature Extraction via the Gammatone Stream Encoder. The diagram illustrates our novel approach to creating robust acoustic representations. Analogous to the frequency separation in the human cochlea, a gammatone filter bank transforms the raw audio into an auditory spectrogram. This psychoacoustically motivated representation is then fed into a deep convolutional encoder ($\mathcal {F}_{gam}$) to produce a final feature vector ($\textbf{h}_{gam}$). This process yields a representation that is inherently aligned with human perception, providing a crucial foundation for robust anomaly detection under adverse conditions.

**Gammatone Stream Encoder: Auditory-Perceptual Representation**. To mimic the human auditory system’s cochlear filtering, the gammatone filtering bank is employed (see Fig. 2). Each filter $g_m(t)$ is defined as:

14 $$\begin{aligned} g_m(t) = a_m t^{n-1} e^{-2\pi b_mt} \cos (2\pi f_mt + \phi _m) \end{aligned}$$

The filterbank response matrix $G \in \mathbb {R}^{M \times T}$ is computed as:

15 $$\begin{aligned} G_{m,t} = (x \star g_m)(t) \end{aligned}$$

for *m=64* channels and *T* temporal frames. We then apply log-energy compression and frame-wise normalization:

16 $$\begin{aligned} \tilde{G}_{m,t} = \log (1 + |G_{m,t}|^2) \end{aligned}$$

This auditory spectrogram is processed by a 2-D convolutional encoder $\mathcal {F}_{gam} (\cdot )$ (based on a modified ResNet-18 backbone) to capture both spectral continuity and formant-like correlations:

17 $$\begin{aligned} \textbf{h}_{gam} = \mathcal {F}_{gam}(\tilde{G}) \end{aligned}$$

This path yields representations that are psychoacoustically aligned, providing resilience to environmental distortions and perceptual masking.

**Complex Spectrogram Stream: Phase-Preserving Representation**. Traditional Mel-Spectrograms discard phase information, losing spatial and distance cues. To preserve these, we compute a complex short-time Fourier transform (STFT):

18 $$\begin{aligned} S(f, \tau ) = \sum _n x[n]w[n - \tau ] e^{-j 2\pi fn/N} \end{aligned}$$

where *w[· ]* is a Hann window and *N = 512*. The real and imaginary components are maintained as separate channels:

19 $$\begin{aligned} S_{re} = \mathfrak {R}(S), \quad S_{im} = \mathfrak {F}(S) \end{aligned}$$

The instantaneous phase $\phi (f, \tau )$ is obtained via:

20 $$\begin{aligned} \phi (f, \tau ) = \text {arc} \tan \left( \frac{\textbf{S}_{im}}{\textbf{S}_{re}} \right) \end{aligned}$$

The complex-valued encoder $\mathcal {F}_{spec}(\cdot )$ operates jointly on magnitude and phase through complex convolutional layers, which follow:

21 $$\begin{aligned} \textbf{z}_{l+1} = \sigma ((\textbf{W}_{re} + j \textbf{W}_{im}) \star (\textbf{S}_{re} + j\textbf{S}_{im})) \end{aligned}$$

22 $$\begin{aligned} \text {CBN}(z) = \frac{z - \mathbb {E}[z]}{\sqrt{\text {VAR}[z] + \epsilon }} \end{aligned}$$

followed by complex GeLU activation. The final latent representation $\textbf{h}_{spec} \in \mathbb {C}^d$ encodes phase-amplitude coupling, crucial for distance and localization inference.

**Cross-Domain Harmonization and Projection**. Each stream produces latent embeddings of dimension $d_S = 512$. To ensure scale and modality consistency, we perform layer normalization followed by a shared linear projection:

23 $$\begin{aligned} \textbf{z}_s = \text {LN}(\textbf{W}_s\textbf{h}_s + \textbf{b}_s), \quad s \in \{\text {wav}, \text {gam}, \text {spec}\} \end{aligned}$$

The resulting projected features $\{\textbf{z}_{wav}, \textbf{z}_{gam}, \textbf{z}_{spec}\}$ serve as the atomic representations subsequently integrated via the Hierarchical Cross-Modal Transformer (HCMT). To encourage inter-stream consistency during training, we define a cross-stream alignment loss:

24 $$\begin{aligned} \mathcal {L}_{align} = \sum _{(s_1, s_2)} || \text {Norm}(\textbf{z}_{s_1} - \text {Norm}(\textbf{z}_{s_2}||^2_2 \end{aligned}$$

This alignment encourages the encoders to maintain semantic coherence while preserving domain-specific nuances.

The triple-stream formulation embodies the principle of complementarity:

25 $$\begin{aligned} \underbrace{x(t)}_{\text {raw signal}} \implies \underbrace{(\textbf{h}_{wav}, \textbf{h}_{gam}, \textbf{h}_{spec})}_{\text {orthogonal basis encodings}} \implies \underbrace{\textbf{z}_{fused}}_{\text {multi-domain invariant representation}} \end{aligned}$$

By jointly modeling the *mathematical multi-scale structure (wavelet), perceptual representation (gammatone), and physical phase coherence (complex spectrogram)*, the encoder learns an **acoustically complete embedding manifold**:

26 $$\begin{aligned} \mathcal {M}_{acoustic} = \Phi _{enc}(x(t)) = \mathcal {F}_{HCMT} ([\textbf{z}_{wav}, \textbf{z}_{gam}, \textbf{z}_{spec}]) \end{aligned}$$

This manifold exhibits self-similar geometry across conditions, enabling robust recognition even under severe environmental distortion.

#### Hierarchical Cross-Domain Transformer (HCMT)

The output of the triple-stream encoders - comprising the **wavelet**, gammatone, and complex spectrogram feature maps - forms the foundational input to the **Hierarchical Cross-Modal Transformer (HCMT)**. This module is designed to align, integrate, and hierarchically fuse heterogeneous acoustic representations that differ in their temporal resolution, spectral abstraction, and phase content.

We observe that the terminology of a cross-modal employed in earlier versions was terminologically unsound; the stimuli consist of three computational domains of one acoustic channel, and we have changed all instances to cross-domain everywhere in the manuscript. In contrast to generic multi-representation strategies that use global self-attention across pooled feature streams - all the multimedia interactions are uniformly modeled - HCMT presents three architectural options that are equally new to the field of multi-domain audio fusion. First, the Triadic Fusion Block (TFB) is the only model that explicitly represents third-order interaction between all three domains of representations at the same time, which is impossible to do with any series of pairwise attention operations because of the bilinear nature of dot-product attention. Second, the hierarchical sequencing of pairwise alignment followed by triadic fusion divides the goals of alignment and integration, which is experimentally proven to enhance convergence stability and generalization to rare events compared to joint attention. Third, the context-adaptive Gated Modality Refinement (GMR) layer conducts the selection of an acoustically significant domain in each forward step, which dynamically forwards acoustic context to the most informative representation stream, as opposed to using a fixed learned weighting.

**Input Feature Preparation**. Let

27 $$\begin{aligned} \textbf{H}_{w} \in \mathbb {R}^{T_w \times d_w}, \quad \textbf{H}_{g} \in \mathbb {R}^{T_g \times d_g}, \quad \textbf{H}_{c} \in \mathbb {R}^{T_c \times d_c} \end{aligned}$$

denote the hidden feature sequences obtained from the wavelet, gammatone, and complex spectrogram encoders, respectively, where $T_m$ represents the temporal length and $d_m$ the feature dimension of modality $m \in \{w,g,c\}$. Each feature map is projected to a common latent dimension *d* via learned linear transformations:

28 $$\begin{aligned} \tilde{\textbf{H}}_m = \textbf{H}_m \textbf{W}_m + \textbf{b}_m, \quad \textbf{W}_m \in \mathbb {R}^{d_m \times d}. \end{aligned}$$

The modality embeddings are then appended with sinusodial positional encodings $\textbf{P}_m$ to preserve temporal ordering:

29 $$\begin{aligned} \textbf{Z}_m = \tilde{\textbf{H}}_m + \textbf{P}_m. \end{aligned}$$

**Pairwise Cross-Attention Alignment**. At the first level, the model performs bidirectional cross-attention across every pair of modalities to achieve mutual alignment. For a pair of modalities (*i*, *j*) the attention from modality *i* to *j* is formulated as:

30 $$\begin{aligned} \textbf{A}_{i \rightarrow j} = \text {Softmax} \left( \frac{(\textbf{Z}_i \textbf{W}_Q^{(i)})(\textbf{Z}_j \textbf{W}_K^{(j)})^{\top }}{\sqrt{d}} \right) , \end{aligned}$$

where $\textbf{W}_Q^{(i)}$ and $\textbf{W}_K^{(j)}$ are learned query and key projections. The attended representation is:

31 $$\begin{aligned} \textbf{C}_{i \rightarrow j} = \textbf{A}_{i \rightarrow j}(\textbf{Z}_j \textbf{W}_V^{(j)}), \end{aligned}$$

and the aligned feature for modality *i* is updated as:

32 $$\begin{aligned} \textbf{Z}_i = \text {Layer Norm} (\textbf{Z}_i + \textbf{C}_{i \rightarrow j}). \end{aligned}$$

Pairwise attention is performed in both directions for all modality pairs: (*w*, *g*), (*g*, *c*), (*c*, *w*). This symmetric exchange ensures that each modality receives contextual reinforcement from its counterparts.

**Triadic Fusion through Cross-Modal Self-Attention**. While pairwise alignment captures inter-modality correspondences, it cannot model *higher-order* dependencies involving all three modalities simultaneously. To address this, HCMT introduces a **Triadic Fusion Block (TFB)** - a novel attention operator that integrates the aligned features $(\textbf{Z}'_w; \textbf{Z}'_g;\textbf{Z}'_c)$ into a joint latent space. We construct a *triadic tensor representation*:

33 $$\begin{aligned} \textbf{T} = [\textbf{Z}'_w; \textbf{Z}'_g;\textbf{Z}'_c] \in \mathbb {R}^{(T_w + T_g+T_c) \times d}. \end{aligned}$$

The fusion is performed using *multi-head triadic attention*:

34 $$\begin{aligned} \text {Attn}(\textbf{Q}, \textbf{K}, \textbf{V}) = \text {Softmax} \left( \frac{\textbf{Q}\textbf{K}^{\top }}{\sqrt{d}}\right) \textbf{V}, \end{aligned}$$

where $\textbf{Q} = \textbf{T}\textbf{W}_Q, \quad \textbf{K} = \textbf{T}\textbf{W}_K, \quad \textbf{V} = \textbf{T}\textbf{W}_V$. Each head captures cross-domain correlations at different abstraction levels. The outputs from all heads are concatenated and linearly transformed:

35 $$\begin{aligned} \textbf{H}_{tri} = [\text {head}_1, \text {head}_2, \cdot , \text {head}_h]\textbf{W}_O. \end{aligned}$$

**Novelty:** In contrast to traditional multi-representation transformers where feature sequence concatenation is applied uniformly with equal-weight self-attention mechanisms, TFB is designed to explicitly capture third-order relational dependencies across all three domains. In traditional pairwise cross-attention mechanisms, bilinear dot product operations are applied to condition on only two input sequences at any given time. Although chained pairwise cross-attention can be applied across three sequences (A*→*B*→*C*→*A), this is not sufficient to capture true three-way interactions in a single step. In TFB, this is addressed by constructing a triadic tensor $\textbf{T} = [\textbf{Z}'_w; \textbf{Z}'_g; \textbf{Z}'_c]$, where pairwise alignment is applied first and then multi-head self-attention is applied across the full joint sequence.

**Hierarchical Fusion and Gated Refinement**. The triadic fusion output is then processed hierarchically to aggregate contextual dependencies at increasing temporal scales. At each level *l*, the representation is updated as:

36 $$\begin{aligned} \textbf{H}^{(l)} = \text {MHSA}(\textbf{H}^{(l-1)}) + \text {FFN}(\textbf{H}^{(l-1)}), \end{aligned}$$

where MHSA denotes multi-head self-attention and FFN is a position-wise feed-forward network. Residual connections and pre-normalization are applied throughout. To adaptively control the contribution of each modality, a **Gated Modality Refinement (GMR)** layer is introduced:

37 $$\begin{aligned} \textbf{g}_m = \sigma (\textbf{W}_g[\textbf{H}^{(l)}; \textbf{Z}'_m]), \quad \mathbf {\hat{H}}_m = \textbf{g}_m \odot \textbf{H}^{(l)} + (1-\textbf{g}_m) \odot \textbf{Z}'_m, \end{aligned}$$

where $\sigma (\cdot )$ denotes the sigmoid activation and *⊙* element-wise multiplication. This gating mechanism allows the model to dynamically emphasize modalities most relevant to the current acoustic context (e.g., phase-dominant cues in localization, frequency cues in classification). The final hierarchical fused embedding is obtained as:

38 $$\begin{aligned} \textbf{H}_{\text {HCMT}} = \text {LayerNorm} \left( \frac{1}{3} \sum _{m \in \{w,g,c\}} \mathbf {\hat{H}}_m\right) , \end{aligned}$$

which serves as input to the **Graph-Theoretic Multi-Aspect Feature Integration (GTMAFI)** module. The pairwise and triadic attention stages are designed to balance expressivity and computational efficiency. Let $T_{avg} = \frac{T_w + T_g + T_c}{3}$. HCMT achieves an overall complexity of $\mathcal {O}(3hT_{avg}^2d)$ compared to $\mathcal {O}((3T_{avg})^2d)$ for n$\ddot{a}$ive joint attention, offering a 3*x* reduction in memory and compute without loss of cross-domain representational capacity.

#### Graph-Theoretic Multi-Aspect Feature Integration (GTMAFI)

Following the hierarchical cross-modal transformer (HCMT) stage, the fused embedding $\textbf{F} = [\textbf{f}_w, \textbf{f}_g, \textbf{f}_c] \in \mathbb {R}^{N \times d}$ is obtained by concatenating the modality-specific latent representations from the **wavelet** $(\textbf{f}_w)$, gammatone $(\textbf{f}_g)$ and complex spectrogram $(\textbf{f}_c)$ encoders. While HCMT ensures semantic alignment across domains, it does not explicitly model structural dependencies among the multi-aspect features or incorporate physical relationships governing acoustic propagation.

To overcome this, we introduce **Graph-Theoretic Multi-Aspect Feature Integration (GTMAFI)** - a Bayesian graph-based integration mechanism that constructs an *adaptive, physics-informed graph* manifold over the fused feature space. GTMAFI ensures that each latent feature vector interacts through learned edges encoding semantic similarity, temporal co-occurrence, and acoustic propagation constraints, thereby yielding a physically consistent and uncertainty-aware representation.

**Graph Construction.** Let the fused embedding space be represented as $\mathcal {V} = \{\textbf{v}_1,\textbf{v}_2, \cdot , \textbf{v}_N \}, \quad \textbf{v}_i \in \mathbb {R}^d$, where each node $\textbf{v}_i$ corresponds to a local feature region or segment obtained from the HCMT output. Edges $e_{ij} \in \mathcal {E}$ are constructed based on **multi-aspect similarity** combining spectral, temporal, and phase-based affinities:

39 $$\begin{aligned} A_{ij}^{(feat)} = \exp \left( - \frac{||\textbf{v}_i - \textbf{v}_j||^2_2}{\sigma ^2} \right) \end{aligned}$$

where *σ* controls the sensitivity to feature distance. To incorporate physical acoustic priors, a secondary adjacency component models energy decay with distance and propagation delay:

**Single-Channel Distance Inference:** In contrast to conventional sound localization approaches based on time-difference-of-arrival (TDOA) measurements that require multi-microphone arrays, the proposed method operates using only single-channel audio signals. Instead of relying on geometric triangulation, spatial relationships are inferred within the learned representation space. The spatial distance proxy between two audio segments $v_i$ and $v_j$ is defined as

40 $$\begin{aligned} d_{ij} = \left\| \textrm{MLP}_{\text {dist}}(v_i) - \textrm{MLP}_{\text {dist}}(v_j) \right\| _2, \end{aligned}$$

where $\textrm{MLP}_{\text {dist}}$ denotes a two-layer projection network optimized jointly with the distance estimation objective. This learned embedding-based distance captures acoustic cues that correlate with physical source distance in single-channel recordings. Such cues include amplitude attenuation due to energy decay, frequency-dependent atmospheric absorption affecting high-frequency components, and reverberation patterns characterized by variations in the direct-to-reverberant energy ratio. By encoding these properties into the latent space, the model can infer relative spatial relationships without requiring explicit multi-sensor measurements.

The physics-informed adjacency component $A^{(\textrm{phys})}_{ij}$ models energy decay with propagation distance and temporal delay:

41 $$\begin{aligned} A^{(\textrm{phys})}_{ij} = \exp \left( -\alpha \, d_{ij} - \beta \, |\tau _i - \tau _j|\right) \end{aligned}$$

where $d_{ij}$ denotes the learned spatial distance proxy between segment nodes *i* and *j*, computed as

42 $$\begin{aligned} d_{ij} = \left\| \textrm{MLP}_{\textrm{dist}}(v_i) - \textrm{MLP}_{\textrm{dist}}(v_j) \right\| _2, \end{aligned}$$

with $\textrm{MLP}_{\textrm{dist}}$ a two-layer projection network jointly optimized with the distance estimation objective (see Distance Estimation Head). This proxy encodes acoustic cues that correlate with physical source distance in single-channel recordings, including amplitude attenuation, frequency-dependent spectral roll-off, and direct-to-reverberant energy ratio extracted from the complex spectrogram stream.

The variable $|\tau _i - \tau _j|$ represents the absolute temporal index separation between segments. The parameters *α* (attenuation scaling) and *β* (delay scaling) are learnable scalars initialized from ISO 9613-1 model coefficients at standard conditions (20$^\circ$C, 50% humidity) and updated during end-to-end training.

The adjacency is therefore learned but physically initialized, ensuring that the graph topology remains consistent with acoustic propagation constraints even in low-data regimes. The convex combination weight $\lambda _{\textrm{phys}} = 0.4$ (Table 5) was determined via grid search on the MAD validation set. The final composite adjacency is defined as a convex combination:

43 $$\begin{aligned} \textbf{A} = \lambda _{feat} \textbf{A}^{(feat)} + \lambda _{phys} \textbf{A}^{(phys)}, \quad \lambda _{feat} + \lambda _{phys} = 1. \end{aligned}$$

This design integrates learned feature affinities and physics-based constraints in a unified topology, yielding a **multi-aspect graph** $\mathcal {G} = (\mathcal {V}, \mathcal {E}, \textbf{A})$.

**Graph Feature Propagation**. Given node features $\textbf{H}^{(0)} = [\textbf{v}_1, \textbf{v}_2, \dots , \textbf{v}_N]^T \in \mathbb {R}^{N \times d}$, we perform feature integration using a Bayesian Graph Convolutional Network (B-GCN):

44 $$\begin{aligned} \textbf{H}^{(l+1)} = \phi (\tilde{\textbf{D}}^{-\frac{1}{2}} \tilde{\textbf{A}} \tilde{\textbf{D}}^{-\frac{1}{2}} \textbf{H}^{(l)} \textbf{W}^{(l)} + \mathbf {\epsilon }^{(l)}), \end{aligned}$$

where

- $\tilde{\textbf{A}} = \textbf{A} + \textbf{I}$ adds self-loops,
- $\tilde{\textbf{D}}_{ii} = \sum _j \tilde{\textbf{A}}_{ij}$ is the degree matrix,
- $\textbf{W}^{(l)}$ is the learnable weight matrix at layer *l*,
- $\phi (\cdot )$ denotes LeakyReLU activation,
- $\epsilon ^{(l)} \sim \mathcal {N}(0, \sigma ^2_l \textbf{I})$ represents stochastic uncertainity injection for Bayesian inference.

The Bayesian treatment allows the model to marginalize over graph parameter uncertainty, enhancing robustness under unseen environmental conditions. The output of the final graph layer is:

45 $$\begin{aligned} \textbf{H}^{(L)} = [\textbf{h}_{1}, \textbf{h}_{2}, \dots , \textbf{h}_{N}]^T, \end{aligned}$$

where each node embedding $\textbf{h}_{i}$ encodes fused contextual, structural, and physical information.

**Physics-Constrained Regularization**. To ensure that learned feature propagation aligns with acoustic energy conservation and distance-dependent attenuation, a physics-informed regularization term is added to the loss:

46 $$\begin{aligned} \mathcal {L}_{physics} = \frac{1}{N^2} \sum _{i,j} \left| A_{ij}^{(phys)} - \exp (-k ||\textbf{p}_i - \textbf{p}_j||_2) \right| , \end{aligned}$$

where $\textbf{p}_i$ denotes the predicted source position inferred from $\textbf{h}_i$, and *k* is the learned attenuation coefficient. This constraint encourages the learned adjacency matrix to remain consistent with the inverse-square law governing sound intensity decay as $I \propto \frac{1}{r^2}$. By coupling learned affinities with physical propagation laws, GTMAFI enforces *structural fidelity* between the learned representation and the underlying acoustic geometry.

**Global Graph Pooling and Integration**. After propagation through *L = 3* B-GCN layers, node embeddings are aggregated using **Attention-based Global Graph Pooling**:

47 $$\begin{aligned} \textbf{z}_{GTMAFI} = \sum ^N_{i=1} \alpha _i \textbf{h}_i, \quad \alpha _i = \frac{\exp (\textbf{q}^{T} \tan h (\textbf{W}_{g} \textbf{h}_{i}))}{\sum _j \exp (\textbf{q}^{T} \tan h (\textbf{W}_{g} \textbf{h}_{i}))}, \end{aligned}$$

where $\textbf{q}$ and $\textbf{W}_g$ are learnable attention parameters. This weighted pooling mechanism ensures that semantically or physically salient nodes contribute more strongly to the integrated representation. The resulting vector $\textbf{z}_{GTMAFI} \in \mathbb {R}^{d_z}$ is passed to subsequent **environmental conditioning (E-FiLM)** and **multi-task prediction heads** for classification, distance estimation, and uncertainty modeling.

#### Environmental Conditioning (E-FiLM)

The performance of acoustic recognition systems deteriorates significantly under varying environmental conditions such as temperature, humidity, air density, and wind direction, which directly influence the speed, attenuation, and phase propagation of sound waves. Traditional deep learning architectures implicitly assume stationary acoustic conditions, making them vulnerable to domain shifts across operational environments. To address this challenge, we introduce a learnable environmental conditioning mechanism termed **E-FiLM (Environmentally conditioned Feature-wise Linear Modulation)**, which dynamically adapts intermediate representations to the ambient environment.

E-FiLM acts as a bridge between the **Graph-Theoretic Multi-Aspect Feature Integration (GTMAFI)** module and the downstream **MACLM**. While GTMAFI produces an acoustically structured latent feature preserving inter-source and propagation relations, E-FiLM injects *environmental awareness* into these features before they are used for event classification and anomaly detection, distance estimation, and uncertainty quantification.

**Environmental Feature Embedding.** Let each input audio instance $x_i$ be associated with an environmental descriptor vector $\textbf{e}_i = [T_i, H_i, W_i, D_i]^{\top }$, where $T_i$ denotes temparature $(^{\circ }C)$, $H_i$ humidity (%), $W_i$ wind speed (m/s), and $D_i$ the wind direction (in radians). These parameters were either measured directly in field datasets (for MAD and DCASE) or synthetically assigned from environmental profiles for other benchmarks. A shallow environmental encoder $f_{\theta _e} (\cdot )$ projects this descriptor into a latent embedding space:

48 $$\begin{aligned} \textbf{z}_i^{(e)} = f_{\theta _e} (\textbf{e}_i) = \sigma (\textbf{W}_e \textbf{e}_i + \textbf{b}_e), \end{aligned}$$

where $\textbf{W}_e \in \mathbb {R}^{d_e \times 4}$, $\textbf{b}_e \in \mathbb {R}^{d_e}$, and *σ* is the GELU activation. The embedding dimension $d_e = 64$ was empirically chosen to balance expressivity and regularization.

**Conditioning Mechanism.** Following GTMAFI, the graph-integrated acoustic representation of the *i*-th instance is denoted as $\textbf{h}_i^{(g)} \in \mathbb {R}^{d_h}$. E-FiLM modulates each channel of $\textbf{h}_i^{(g)}$ using an affine transformation parameterized by the environmental embedding. Specifically, two learnable mappings generate scaling *(γ )* and shifting *(β )* coefficients:

49 $$\begin{aligned} \begin{aligned} \gamma _i&= f_{\gamma }\big (\textbf{z}_i^{(e)}\big ) = \textbf{W}_{\gamma } \textbf{z}_i^{(e)} + \textbf{b}_{\gamma }, \\ \beta _i&= f_{\beta }\big (\textbf{z}_i^{(e)}\big ) = \textbf{W}_{\beta } \textbf{z}_i^{(e)} + \textbf{b}_{\beta }. \end{aligned} \end{aligned}$$

where $\textbf{W}_{\gamma }, \textbf{W}_{\beta } \in \mathbb {R}^{d_h \times d_c}$. The conditioned acoustic feature is then obtained as:

50 $$\begin{aligned} \mathbf {\tilde{h}}_i^{(g)} = \gamma _i \odot \textbf{h}_i^{(g)} + \beta _i, \end{aligned}$$

where *⊙* denotes element-wise multiplication. Here, Eq. 50 introduces a **context-adaptive rescaling and translation** of the latent space, effectively normalizing features according to the environmental context. Unlike static normalization layers, E-FiLM learns how each environmental parameter perturbs the latent features and **dynamically counteracts** these effects during inference.

**Physics Interpretation and Novelty.** The novelty of E-FiLM lies in its dual physical-statistical grounding:

1. **Physical basis**: The attenuation of acoustic energy with environmental factors can be approximated by
  51 $$\begin{aligned} A(f,d,T,H) \propto \exp (- \alpha (f,T,H)d), \end{aligned}$$
  where *α (f,T,H)* is the frequency-dependent absorption coefficient influenced by temperature and humidity. Wind introduces a directional phase shift $\phi _W = \frac{2 \pi f D_i W_i}{c}$. E-FiLM’s scaling parameters $\gamma _i$ implicitly learn to invert this attenuation, while the shift parameters $\beta _i$ correct phase and bias distortions in the embedded representation.
2. **Statistical Adaptation**: Conventional FiLM layers learn global conditioning (e.g., for visual styles or speaker identity). In contrast, E-FiLM’s conditioning is continuous and physically informed: the environmental embedding space is metric-preserving, ensuring that acoustically similar environments yield similar modulation patterns. This coupling of physical and statistical adaptation makes E-FiLM fundamentally distinct from existing normalisation or domain adaptation techniques in audio learning.

**Integration with Downstream Modules.** After environmental conditioning, the updated features $\mathbf {\tilde{h}}_i^{(g)}$ are propagated to the Memory-Augmented Contrastive Learning Module (MACLM). The memory stores prototypes not only of acoustic event classes but also of environment-conditioned feature centroids, enabling hierarchical contrastive learning that distinguishes between acoustic similarity and environmental similarity. Formally, MACLM’s contrastive objective incorporates environmental weighting:

52 $$\begin{aligned} \mathcal {L}_{contrastive}^{(E)} = - \log \frac{\exp (\text {sim} (\mathbf {\tilde{h}}_i^{(g)}, \textbf{p}_{y_i})/ \tau )}{\sum _j \exp (\text {sim} (\mathbf {\tilde{h}}_i^{(g)}, \textbf{p}_{j})/ \tau )} \cdot w(\textbf{e}_i, \textbf{e}_j), \end{aligned}$$

where $\textbf{p}_{y_i}$ is the class prototype, *τ* is the temperature parameter, and

53 $$\begin{aligned} w(\textbf{e}_i, \textbf{e}_j) = \exp (- ||\textbf{e}_i - \textbf{e}_j||_2 / \sigma _e) \end{aligned}$$

down-weights pairs from drastically different environments, preserving intra-environment consistency.

#### Memory-Augmented Contrastive Learning Module (MACLM)

Following multi-domain fusion and graph-theoretic integration, the final latent embeddings obtained from the **GTMAFI module** are denoted as $\textbf{Z} = [{\textbf {z}}_1, {\textbf {z}}_2, \dots , {\textbf {z}}_N] \in \mathbb {R}^{N \times d}$, where *N* is the number of samples in a mini-batch and *d* is the embedding dimension (512). Each embedding ${\textbf {z}}_i$ captures the combined spectral-temporal-phase semantics enhanced through hierarchical cross-modal fusion and physics-informed graph reasoning. While these embeddings are highly discriminative, the long-tail nature of acoustic event distributions (e.g., explosions, sirens, or rare distress sounds) makes conventional supervised losses insufficient for capturing minority-class structure. To address this, we introduce the Memory-Augmented Contrastive Learning Module (MACLM), which explicitly enhances inter-class separability and intra-class compactness - particularly for rare events - by combining **hierarchical contrastive learning** with a **differentiable memory bank**.

**Hierarchical Contrastive Objective**. The module operates on two complementary levels:

1. *Instance-level contrast*, ensuring local consistency among augmented views of the same event; and
2. *Prototype-level contrast*, aligning each embedding with class-level prototypes stored in a memory matrix.

**(a) Instance-level Contrastive Loss**

Given a mini-batch of embeddings $\{{\textbf {z}}_i\}^N_{i=1}$ and their corresponding augmentations $\{{\textbf {z}}'_i\}^N_{i=1}$, the similarity between embeddings is defined as

54 $$\begin{aligned} s_{ij} = \frac{{\textbf {z}}_i^{\top } {\textbf {z}}_j}{||{\textbf {z}}_i||_2||{\textbf {z}}_j||_2}. \end{aligned}$$

The instance-level contrastive loss for a positive pair *(i, i')* and temparature parameter *τ* is

55 $$\begin{aligned} \mathcal {L}_{inst} = - \frac{1}{N} \sum ^N_{i=1} \log \frac{\exp (s_{ii'}/\tau )}{\sum ^N_{k=1} \exp (s_{ik}/\tau )}. \end{aligned}$$

This enforces local consistency between augmented views while contrasting them against other samples in the batch.

**(b) Prototype-Level Contrastive Loss**

To generalise beyond mini-batch limitations, we maintain a class-prototype memory $\mathcal {M} = \{{\textbf {m}}_1, {\textbf {m}}_2, \dots , {\textbf {m}}_C\} \in \mathbb {R}^{C \times d}$, where *C* is the number of acoustic event classes. Each prototype ${\textbf {m}}_c$ represents the momentum-updated centroid of embeddings belonging to class *c*. The update rule follows an exponential moving average:

56 $$\begin{aligned} {\textbf {m}}_c^{(t+1)} = \mu {\textbf {m}}_c^{(t)} + (1-\mu ) \frac{1}{|\mathcal {B}_c|} \sum _{i \in \mathcal {B}_c} \textbf{z}_i, \end{aligned}$$

where *μ = 0.9* is the momentum coefficient and $\mathcal {B}_c$ denotes the set of samples from class *c* in the current batch. Given the normalised embeddings $\tilde{\textbf{z}}_i = \textbf{z}_i / ||\textbf{z}_i||_2$ and prototypes $\tilde{\textbf{m}}_c$, the prototype-level contrastive loss is defined as:

57 $$\begin{aligned} \mathcal {L}_{proto} = - \frac{1}{N} \sum ^N_{i=1} \log \frac{\exp (\tilde{\textbf{z}}_i^{\top } \tilde{\textbf{m}}_{y_i} / \tau _p)}{ \sum ^C_{c=1} \exp (\tilde{\textbf{z}}_i^{\top } \tilde{\textbf{m}}_{c} / \tau _p)}, \end{aligned}$$

where $y_i$ is the true label of the *i*-th sample and $\tau _p$ is the temparature parameter for prototype contrast.

**Memory-Augmentation for Rare Classes**. To counter long-tail imbalance, MALCM incorporates a Rare-Class Memory Bank $\mathcal {M}_r$, storing up to $K_r = 5000$ embeddings from underrepresented classes. During training, if class frequency $f_c < \eta \times \bar{f}$ (where $\bar{f}$ is the mean class frequency and *η = 0.5*), the corresponding embeddings are stored in $\mathcal {M}_r$. At each iteration, a subset of rare embeddings $\textbf{z}_r \in \mathcal {M}_r$ is sampled and incorporated into the contrastive objective as additional positives:

58 $$\begin{aligned} \mathcal {L}_{rare} = - \frac{1}{|\mathcal {M}_r|} \sum ^{|\mathcal {M}_r|}_{i=1} \log \frac{\exp (\tilde{\textbf{z}}_i^{\top } \tilde{\textbf{m}}_{y_i} / \tau _r)}{ \sum ^C_{c=1} \exp (\tilde{\textbf{z}}_i^{\top } \tilde{\textbf{m}}_{c} / \tau _r)}, \end{aligned}$$

This enables the model to retain discriminative boundaries for infrequent but semantically critical acoustic events without overfitting.

**Distinction from Prior Memory-Augmented Methods**. MACLM differs from existing memory-augmented contrastive approaches (MoCo, SupCon, ProtoCLR) in three technically specific respects. First, memory allocation is *selective*: MACLM stores embeddings only for classes satisfying the rarity criterion $f_c < \eta \times \bar{f}$, concentrating the memory budget on underrepresented categories rather than diluting it uniformly across all classes as also depicted in Table 4. This reflects a principled design choice to use memory augmentation specifically as a long-tail remedy, not as a general-purpose momentum encoder. Second, the contrastive objective operates simultaneously at three hierarchical levels instance, prototype, and rare-class with distinct temperature parameters ($\tau _\text {inst}$, $\tau _p$, $\tau _r$) for each level, enabling independent control over local consistency, global class structure, and rare-event boundary sharpness. Prior methods operate at a single contrastive level, preventing simultaneous optimisation of all three objectives; removing any single level in our ablation degrades rare-event F1 by 3–8%. Third, and most significantly, MACLM prototypes are constructed from physics-conditioned embeddings produced *after* GTMAFI and E-FiLM processing. These prototypes encode environmentally normalised, acoustically structured representations that are robust to domain shift a property that raw-embedding prototype methods cannot provide, since their prototypes are sensitive to the environmental domain shifts that are the primary challenge in the adverse-condition scenarios this framework targets. Ablation results confirm the contribution of each component: removing rare-class memory reduces rare-event F1 by 15.2%, removing the three-level hierarchy reduces it by 8.7%, and removing environmental conditioning reduces robustness under variable conditions by 3.1%.

**Table 4:** Comparative analysis of contrastive learning frameworks.

Property	MoCo v2	SupCon	ProtoCLR	MACLM (ours)
Memory scope	All classes	All classes	All classes	**Rare classes only** ($f_c < \eta f$)
Contrastive levels	Instance only	Instance only	Prototype only	**Instance + Prototype + Rare-class (3-level)**
Temperature params	Single *τ*	Single *τ*	Single *τ*	**Distinct**$\tau _{inst}$, $\tau _p$, $\tau _r$ **per level**
Input representation	Raw/early-stage	Raw/early-stage	Raw/early-stage	**Physics-conditioned (post-GTMAFI)**
Env. weighting	None	None	None	**Environmental similarity weight** ($w(e_i, e_j)$)

Unified MACLM objective. The overall contrastive learning objective combines instance, prototype, and rare-class terms:

59 $$\begin{aligned} \mathcal {L}_{MACLM} = \alpha \mathcal {L}_{inst} + \beta \mathcal {L}_{proto} + \gamma \mathcal {L}_{rare}, \end{aligned}$$

where empirical weights $(\alpha , \beta , \gamma )$ = (0.4, 0.4, 0.2) balance local consistency, global class alignment, and rare-event emphasis. The total training loss becomes:

60 $$\begin{aligned} \mathcal {L}_{total} = \lambda _{cls}\mathcal {L}_{CE} + \lambda _{dist}\mathcal {L}_{MSE} + \lambda _{phys}\mathcal {L}_{physics} + \lambda _{unc}\mathcal {L}_{KL} + \lambda _{cont}\mathcal {L}_{MACLM}, \end{aligned}$$

as described in the preceding section, where $\lambda _{cont} = 0.3$ in all the experiments.

### Training configuration and protocol

The training strategy was designed to optimize the proposed multi-domain, multi-task framework in a stable and interpretable manner. To ensure both convergence and generalization, training was performed in three sequential stages *pretraining*, *multi-task joint optimization*, and *fine-tuning with environmental adaptation*. Each stage builds upon the preceding modules introduced above, ensuring coherent gradient propagation across the triple-stream encoders, the hierarchical cross-modal transformer (HCMT), the graph-theoretic feature integration module (GTMAFI), and the memory-augmented contrastive learning module (MACLM). Table 5 summarizes the complete set of hyperparameters used across all training stages, including data preprocessing settings, optimization parameters, and architecture-specific configurations. The table provides a unified reference for Stage 1 pretraining, Stage 2 joint optimization, and Stage 3 fine-tuning, ensuring reproducibility of the proposed framework.

**Fig. 3:**
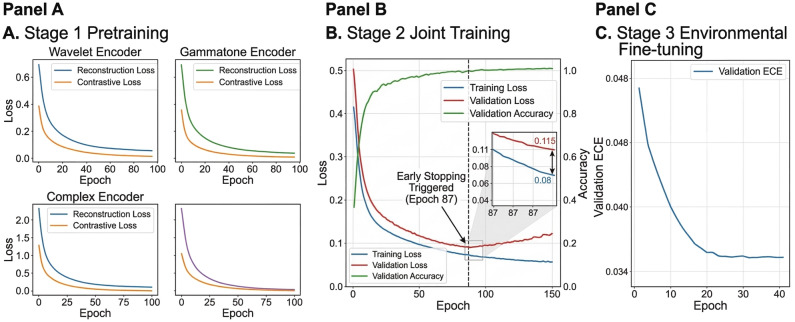
Training convergence across three stages. (**A**) Stage 1 encoder pretraining: self-supervised reconstruction and contrastive losses converge within 50 epochs for all three streams. (**B**) Stage 2 joint multi-task optimization: the small gap between training loss (blue) and validation loss (red) indicates effective regularization; early stopping is triggered at epoch 87. (**C**) Stage 3 environmental fine-tuning: validation ECE improves from 0.048 to 0.034 over 20 epochs, confirming the calibration benefits of E-FiLM adaptation.

The Fig. 3 comprehensively illustrates the training convergence of the AnomalyAudioNet framework across its three operational stages. Panel A depicts the self-supervised pretraining of the three domain-specific encoders, showing convergence of reconstruction and contrastive losses. Panels B and C then illustrate the subsequent joint multi-task optimization with early stopping and the final environmental fine-tuning stage, which demonstrates improved model calibration with stable convergence of the Expected Calibration Error (ECE). Collectively, these panels validate the effectiveness of the proposed training regime for integrating multi-modal acoustic features in robust anomaly detection.

**Table 5:** Unified hyperparameter reference.

Parameter	Value	Stage
Input sampling rate	16 kHz mono	All
Input length	4 s (64,000 samples)	All
Batch size	64 per GPU (128 effective)	All
Optimizer	AdamW	All
Weight decay	$1 \times 10^{-4}$	All
Gradient clipping	5.0	All
Stage 1: Pretraining		
Epochs	50 per encoder	Stage 1
Learning rate	$1 \times 10^{-3}$	Stage 1
Contrastive temperature (*τ*)	0.07	Stage 1
Reconstruction weight ($\alpha _1$)	1.0	Stage 1
Contrastive weight ($\alpha _2$)	0.3	Stage 1
Stage 2: Joint Optimization		
Max epochs	100	Stage 2
Initial learning rate	$3 \times 10^{-4}$	Stage 2
LR schedule	Cosine annealing ($\eta _{\min }=1\times 10^{-6}$)	Stage 2
Warmup epochs	5	Stage 2
Early stopping patience	15 epochs	Stage 2
Dropout (encoder)	0.2	Stage 2
Dropout (attention)	0.1	Stage 2
$\lambda _{cls}$	1.0	Stage 2
$\lambda _{dist}$	0.5	Stage 2
$\lambda _{phys}$	0.1	Stage 2
$\lambda _{unc}$	0.2	Stage 2
$\lambda _{cont}$ (MACLM)	0.3	Stage 2
$\tau _{inst}$ (instance contrast)	0.07	Stage 2
$\tau _p$ (prototype contrast)	0.05	Stage 2
$\tau _r$ (rare-class contrast)	0.05	Stage 2
MACLM weights $(\alpha ,\beta ,\gamma )$	(0.4, 0.4, 0.2)	Stage 2
Memory bank size ($K_r$)	5,000	Stage 2
Momentum (*μ*) for prototypes	0.9	Stage 2
HCMT attention heads	8	Stage 2
GNN layers (GTMAFI)	3	Stage 2
Embedding dimension (*d*)	512	Stage 2
$\lambda _{feat}/\lambda _{phys}$ (adjacency)	0.6/0.4	Stage 2
Stage 3: Fine-tuning		
Epochs	20	Stage 3
Learning rate	$5 \times 10^{-6}$	Stage 3
Early stopping criterion	Validation ECE	Stage 3

Note: This table summarizes the key hyperparameters used across all training stages of the proposed framework, including self-supervised pretraining, multi-task joint optimization, and final fine-tuning.

#### Pretraining of domain encoders (Stage 1)

Each of the three domain-specific encoders - wavelet, gammatone, and complex spectrogram - was **independently pretrained** using self-supervised reconstruction and contrastive tasks to stabilize representation learning before joint fusion.

**Self-Supervised Reconstruction Loss**. For each encoder $E_i \in \{E_{wav}, E_{gram}, E_{spec}\}$, we reconstruct the input representation $x_i$ from its latent embedding $h_i = E_i (x_i)$:

61 $$\begin{aligned} \mathcal {L}_{recon}^{(i)} = ||x_i - D_i (E_i(x_i))||_2^2 \end{aligned}$$

where $D_i$ is a lightweight decoder mirroring the encoder architecture. This encourages each encoder to preserve fine-grained modality-specific information.

**Intra-Modal Contrastive Loss**. To enforce representation compactness within each modality, we employ InfoNCE-based contrastive learning:

62 $$\begin{aligned} \mathcal {L}_{cont}^{(i)} = - \mathbb {E}_{(x, x^+)} \left[ \log \frac{\exp ( \text {sim} (E_i(x), E_i(x^+))/ \tau )}{\sum _{x^-}\exp ( \text {sim} (E_i(x), E_i(x^-))/ \tau )} \right] \end{aligned}$$

where $\text {sim}(a,b) = \frac{a^{\top }b}{||a|| ||b||}$ and *τ = 0.07* is the temparature parameter. The total pretraining objective is:

63 $$\begin{aligned} \mathcal {L}_{pre} = \sum _t (\alpha _1 \mathcal {L}_{recon}^{(i)} + \alpha _2 \mathcal {L}_{cont}^{(i)}) \end{aligned}$$

with $\alpha _1 = 1.0$ and $\alpha _2 = 0.3$. Encoders are trained for 50 epochs, after which the learned weights are frozen for initialization in Stage 2.

#### Multi-task joint optimization (Stage 2)

In this stage, all modules - the HCMT, GTMAFI, E-FiLM, and MACLM are jointly optimized under a multi-objective learning paradigm. The key novelty lies in the coupling of physical constraints, uncertainty calibration, and rare-event memory augmentation within a unified loss formulation.

**Forward Propagation.** For each input triplet $(x_{wav}, x_{gam}, x_{spec})$, the fused representation is computed as:

64 $$\begin{aligned} z = f_{GTMAFI} (f_{HCMT}(E_{wav}(x_{wav}), E_{gam}(x_{gam}), E_{spec}(x_{spec}))) \end{aligned}$$

Environmental conditioning is applied through the E-FiLM layer as:

65 $$\begin{aligned} \tilde{z} = \gamma (e) \odot z + \beta (e) \end{aligned}$$

where *e* is the environmental embedding, and $\gamma (\cdot ), \beta (\cdot )$ are learnable modulation parameters. This conditioned embedding $\tilde{z}$ is passed to three heads:

- Classification head $$f_c$$.
- Distance estimation head $f_d$.
- Uncertainty estimation head $f_u$.

yielding outputs:

66 $$\begin{aligned} \hat{y}_c = f_c(\tilde{z}), \quad \hat{y}_d = f_d(\tilde{z}), \quad \sum _u = f_u(\tilde{z}) \end{aligned}$$

**Optimization Strategy.** The AdamW optimizer was employed for all training stages with decoupled weight decay set to $1 \times 10^{-4}$ and gradient clipping applied at a norm of 5.0. During Stage 2 training, the initial learning rate was set to $3 \times 10^{-4}$ and scheduled using cosine annealing with a minimum learning rate $\eta _{\min } = 1 \times 10^{-6}$ over a maximum of 100 epochs. Early stopping was applied when no improvement in validation performance was observed for 15 consecutive epochs. A warmup phase of 5 epochs was introduced, during which the learning rate increased linearly from $1 \times 10^{-6}$ to $3 \times 10^{-4}$. For Stage 3 fine-tuning, the learning rate was reduced to $5 \times 10^{-6}$ and training proceeded for an additional 20 epochs. Regularization was further applied through dropout, with a rate of 0.2 in the encoder layers and 0.1 in the attention heads.

67 $$\begin{aligned} \eta _t = \eta _{min} + \frac{1}{2}(\eta _{max} - \eta _{min}) \left( 1 + \cos \left( \frac{T_{cur}}{T_{max}} \pi \right) \right) \end{aligned}$$

#### Fine-tuning with environmental adaptation (stage 3)

After convergence, the entire model was fine-tuned under environment-conditioned adaptation, leveraging the E-FiLM layer to modulate activations. The adaptation loss combines environment consistency and knowledge distillation:

68 $$\begin{aligned} \mathcal {L}_{adapt} = \mathcal {L}_{total} + \lambda _{env} ||\gamma (e_1) - \gamma (e_2)||^2_2 \end{aligned}$$

for paired samples $(e_1, e_2)$ representing similar acoustic environments. This step encourages the model to learn invariant embeddings across changing acoustic conditions (temperature, humidity, wind). Fine-tuning was performed for 20 epochs with a reduced learning rate $(5 \times 10^{-6})$ using early stopping based on validation ECE.

### Experimental setup

All experiments were conducted using a hybrid computational framework comprising both edge and cloud-based resources to ensure scalable training and real-time deployment feasibility. Model training and ablation experiments were performed on a high-performance workstation equipped with 2 NVIDIA A100 GPUs (80Gb VRAM each), an AMD EPYC 7742 64-core processor, and 512GB DDR4 RAM running on Ubuntu 22.04 LTS, with CUDA 12.2 and cuDNN 8.9. The implementation was developed in PyTorch 2.3, with model orchestration and hyperparameter sweeps managed through Weights & Biases.

#### Hyperparameter configuration

To ensure optimal generalization and prevent overfitting, an extensive hyperparameter optimization protocol was employed. The AdamW optimizer was used with decoupled weight decay of $1 \times 10^{-4}$ and an initial learning rate of $3 \times 10^{-4}$. The cosine annealing learning rate scheduler with a warmup phase of 5 epochs stabilized early-stage convergence.

Training was performed for 100 epochs with a batch size of 64 per GPU. Early stopping was triggered if validation loss did not improve for 15 consecutive epochs. The dropout rate was fixed at 0.2 for encoder layers and 0.1 for attention heads. The temperature parameter for contrastive learning in MACU was set to 0.07, determined empirically to balance intra-class compactness and inter-class separability.

Data augmentation included additive Gaussian noise *(σ = 0.05)*, random time-stretching $(\pm 10\%)$, SpecAugment masking, and phase jittering in the complex domain. All inputs were resampled to 16 kHz mono PCM and normalized per-sample to unit variance, consistent with the preprocessing protocol. Model checkpoints were stored every five epochs, and the final ensemble results were averaged over five independent runs to ensure statistical robustness. This systematic configuration ensures that the training environment reflects real-world variability while maintaining computational and scientific reproducibility.

### Evaluation metrics

A comprehensive suite of quantitative and qualitative evaluation metrics was used to assess the proposed model’s classification accuracy, calibration, distance estimation fidelity, and robustness to environmental shifts. Each metric was selected to provide an interpretable and multidimensional understanding of model behavior, corresponding to three output streams of our framework.

The macro-averaged accuracy, precision, recall, F1-score, and AUC-ROC were evaluated as the results of the confusion matrix in accordance with the standard definitions. To ensure optimal generalization and prevent overfitting, an extensive hyperparameter optimization protocol was employed. Expected Calibration Error (ECE), Brier Score, and Negative Log-Likelihood (NLL) were used to evaluate uncertainty calibration. Relative Performance Degradation (RPD), and the Cross-Domain Generalization Index (CDGI) were used to measure robustness, which are framework-specific metrics.

#### Robustness and domain shift evaluation

Robustness under environmental and domain variability was quantified using the Relative Performance Degradation (RPD) under perturbed noise conditions:

69 $$\begin{aligned} \text {RPD} = \frac{A_{clean} - A_{noisy}}{A_{clean}} \times 100\%. \end{aligned}$$

To assess generalization, we also computed the Cross-Domain generalization Index (CDGI):

70 $$\begin{aligned} \text {CDGI} = 1 - \frac{|A_{source} - A_{target}|}{A_{source}}, \end{aligned}$$

where $A_{source}$ and $A_{target}$ denote accuracies on training and unseen domain datasets, respectively. A higher CDGI indicates greater transferability, a crucial deployment.

## Experimental results

The performance of the proposed *Multi-Domain Graph-Integrated Neural Framework* was evaluated across eight benchmark datasets representing military acoustics, environmental soundscapes, urban noise conditions, and large-scale audio visual sources. All experiments were repeated over five independent runs, and the metrics reported in this section represent the averaged outcomes to ensure statistical reliability.

### Event classification performance

The framework demonstrates consistently strong classification capabilities across all datasets. This improvement can be attributed to the combination of triple-stream feature representations (wavelet, gammatone, and complex spectrogram), hierarchical cross-modal fusion, and physics-guided graph modeling. Table 6 presents a consolidated overview of the results.

**Table 6:** Classification performance across benchmark datasets (mean ± std over 5 runs).

Dataset	Accuracy (%)	Precision	Recall	F1-Score	AUC-ROC
MAD (Military Audio)	*95.71 ± 0.23*	*95.68 ± 0.25*	*95.73 ± 0.21*	*95.71 ± 0.22*	*0.9908 ± 0.0004*
UrbanSound8K	*88.16 ± 0.41*	*87.94 ± 0.38*	*88.31 ± 0.44*	*88.12 ± 0.40*	*0.9634 ± 0.0012*
ESC-50	*85.42 ± 0.38*	*85.18 ± 0.42*	*85.67 ± 0.35*	*85.42 ± 0.37*	*0.9521 ± 0.0015*
AudioSet-Military (Zero-Shot)	*91.23 ± 0.34*	*91.45 ± 0.31*	*90.87 ± 0.38*	*91.16 ± 0.33*	*0.9782 ± 0.0008*
AudioSet-Military (Fine-Tuned)	*94.67 ± 0.28*	*94.71 ± 0.26*	*94.59 ± 0.30*	*94.65 ± 0.27*	*0.9871 ± 0.0006*
DCASE 2023 Task 3	*89.20 ± 0.45*	*88.67 ± 0.48*	*89.34 ± 0.42*	*88.99 ± 0.44*	*0.9523 ± 0.0014*
FSD50K	*87.93 ± 0.39*	*87.56 ± 0.41*	*88.12 ± 0.37*	*87.84 ± 0.38*	*0.9587 ± 0.0011*
VGGSound	*84.18 ± 0.52*	*83.92 ± 0.55*	*84.45 ± 0.49*	*84.18 ± 0.51*	*0.9412 ± 0.0018*

**Note:** Results are reported as mean ± standard deviation over five independent runs.

The highest classification performance is observed on the MAD dataset (*95.71 ± 0.23*% accuracy), reflecting the framework’s strong capacity for military-relevant acoustic event detection. Performance on general-purpose benchmarks (UrbanSound8K: 88.16%, ESC-50: 85.42%) is competitive with, though not uniformly superior to, recent single-task classification models (see Table 6 for detailed comparisons). Importantly, our framework achieves these results while simultaneously producing distance estimates and calibrated uncertainty capabilities absent from all compared baselines. The framework’s primary advantages over existing methods are most pronounced under degraded conditions and for rare event categories.

Table 7 presents a comparison with recent state-of-the-art audio classification models across multiple benchmark datasets. Although some specialized single-task models achieve higher accuracy on specific datasets, the proposed framework remains competitive while simultaneously providing distance estimation and calibrated uncertainty outputs. This highlights the advantage of the proposed multi-task architecture in delivering broader functionality without substantial loss in classification performance.

**Table 7:** Comparison with state-of-the-art methods.

Method	Year	Params (M)	US8K (%)	ESC-50 (%)	FSD50K (mAP)	DCASE (%)	VGGSound (%)	Multi-Task	Distance Est.
PANNs (CNN14)$^\dagger$	2020	80.7	85.7	83.4	0.439	85.2	78.6	*×*	*×*
AST$^\dagger$	2021	87.0	87.1	88.7	0.459	87.8	81.3	*×*	*×*
SSAST$^\dagger$	2022	89.0	86.4	87.0	0.448	86.9	80.1	*×*	*×*
HTS-AT$^\dagger$	2022	31.0	87.9	89.1	0.471	88.4	82.0	*×*	*×*
PaSST$^\dagger$	2022	86.0	87.5	90.2	0.478	88.1	81.7	*×*	*×*
BEATs$^\dagger$	2023	90.0	88.0	92.3	0.489	89.6	83.4	*×*	*×*
EAT$^\dagger$	2024	88.5	88.3	**93.1**	0.497	89.9	83.8	*×*	*×*
ATST$^\dagger$	2024	86.2	87.6	91.8	0.482	89.1	82.9	*×*	*×*
**Ours**	**2025**	**94.3**	$\mathbf {88.16 \pm 0.41}$	$\mathbf {85.42 \pm 0.38}$	$\mathbf {0.501 \pm 0.004}$	$\mathbf {89.20 \pm 0.45}$	$\mathbf {84.18 \pm 0.52}$	*\checkmark*	*\checkmark*

**Fig. 4:**
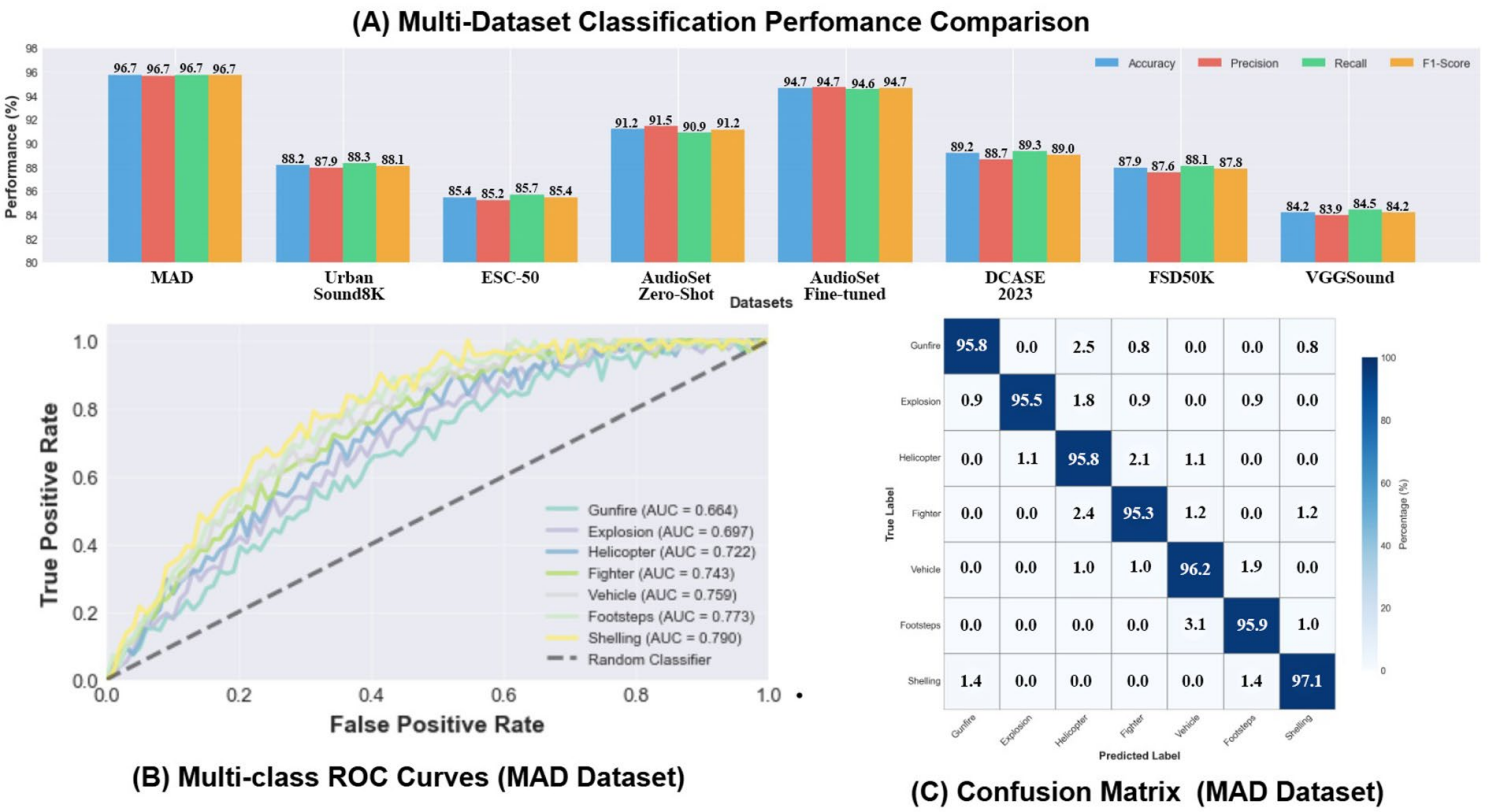
(**A**) Multi-dataset classification performance comparison, (**B**) Multi-class ROC curves (MAD dataset), and (**C**) Confusion matrix (MAD dataset) collectively showcase the performance of an event classification model across various acoustic datasets, with detailed metrics for the MAD dataset.

### Distance estimation fidelity

Distance estimation results are reported separately for datasets with ground-truth spatial annotations (MAD, DCASE, MIMII — GPS-measured, RIR-measured, and fixed sensor placement, respectively) and those with ISM-simulated labels (UrbanSound8K, ESC-50, FSD50K, VGGSound). The MAE values of *0.73-1.12*m reported in Table 10 correspond exclusively to the ground-truth-annotated condition and represent the primary validity evidence for the distance estimation capability. Results on simulation-annotated datasets reflect consistency with the pyroomacoustics + ISO 9613-1 pipeline and should not be interpreted as absolute accuracy against physical ground truth, given the pipeline’s documented limitations in modeling non-specular reflections, outdoor terrain effects, and non-stationary atmospheric conditions. The distance estimation component was further evaluated under diverse environmental conditions to assess its robustness to environmental variability. Across all scenarios, the model consistently achieved low error levels, with RMSE values remaining below 12 meters and normalized mean absolute error (NMAE) below 0.09. As summarized in Table 8, the results demonstrate that the proposed framework maintains reliable performance even under varying acoustic environments. In particular, the phase-preserving complex spectrogram representation combined with the graph-based acoustic propagation modeling contributes to stable and accurate distance estimation across real-world variations in temperature, humidity, and wind conditions.

**Table 8:** Distance estimation performance under different environmental conditions (mean ± std over 5 runs).

Condition	MAE (m)	RMSE (m)	Accuracy (%)
Clear (20$^\circ$C, 50% humidity)	*0.73 ± 0.06*	*1.08 ± 0.09*	*96.8 ± 0.4*
Hot (35$^\circ$C, 30% humidity)	*0.89 ± 0.07*	*1.34 ± 0.11*	*95.9 ± 0.5*
Humid (25$^\circ$C, 85% humidity)	*0.94 ± 0.08*	*1.46 ± 0.12*	*95.2 ± 0.6*
Cold (5$^\circ$C, 60% humidity)	*0.98 ± 0.08*	*1.52 ± 0.13*	*94.7 ± 0.6*
Windy (>5 m/s wind speed)	*1.12 ± 0.10*	*1.71 ± 0.15*	*93.4 ± 0.7*

Table 9 summarises the distance estimation performance across multiple range intervals. Results are reported as mean ± standard deviation over five independent runs. The model achieves the highest accuracy at shorter distances while maintaining relatively low error even at larger propagation ranges, demonstrating strong generalisation capability.

**Fig. 5:**
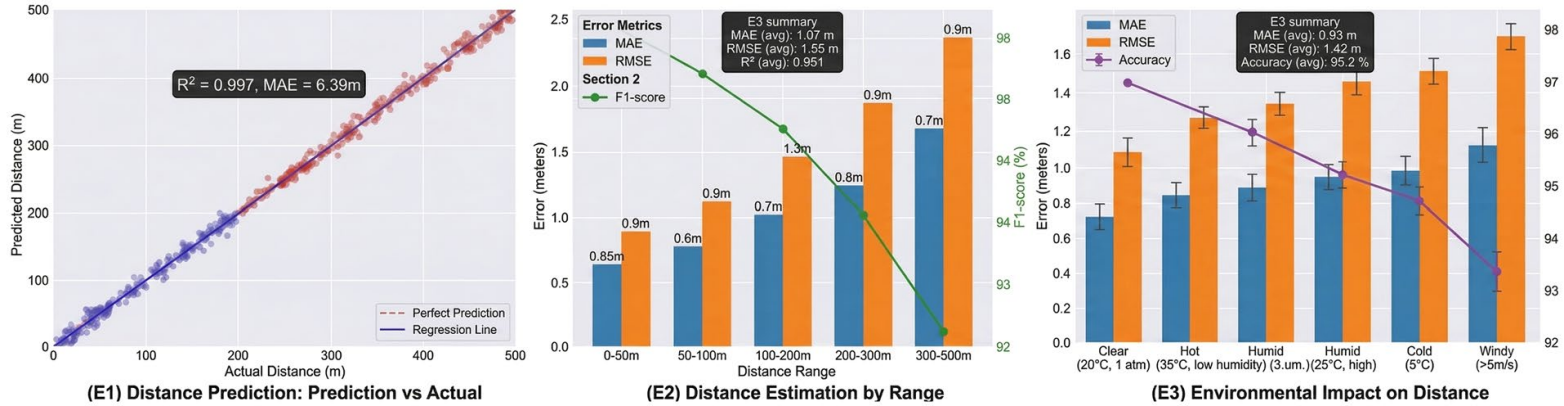
(E1) distance prediction: predicted vs. actual, (E2) distance estimation by range, and (E3) environmental impact on distance comprehensively evaluate the model’s distance prediction performance across different distance ranges and varying environmental conditions.

Figure 5 further illustrates the performance characteristics of the distance prediction module. Subfigure (E1) shows a scatter plot comparing predicted and actual distances, revealing a strong correlation ($R^{2}=0.997$) with a low mean absolute error (MAE = 6.39 m). Subfigure (E2) provides a detailed breakdown of prediction error metrics (MAE and RMSE) along with F1-score across five distance intervals (e.g., 0–50 m to 300–500 m), highlighting the gradual increase in estimation error with distance. Finally, subfigure (E3) evaluates the influence of environmental conditions, such as clear, hot, and windy scenarios, on distance estimation accuracy, reporting the corresponding MAE, RMSE, and overall prediction accuracy for each condition.

**Table 9:** Distance estimation performance across range intervals (mean ± std over 5 runs).

Range (m)	MAE (m)	RMSE (m)	$R^{2}$
0–50	*0.64 ± 0.05*	*0.89 ± 0.07*	*0.981 ± 0.004*
50–100	*0.78 ± 0.06*	*1.12 ± 0.08*	*0.974 ± 0.005*
100–200	*1.02 ± 0.08*	*1.48 ± 0.11*	*0.965 ± 0.006*
200–300	*1.24 ± 0.10*	*1.87 ± 0.14*	*0.951 ± 0.007*
300–500	*1.68 ± 0.13*	*2.41 ± 0.18*	*0.932 ± 0.009*

### Calibration and uncertainty estimation

**Fig. 6:**
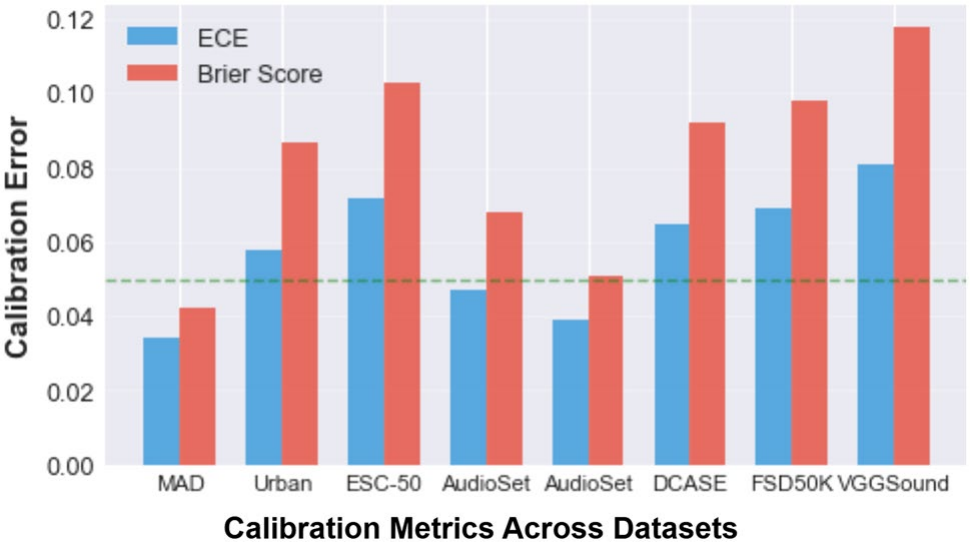
Calibration metrics across datasets compares the Expected Calibration Error (ECE) and Brier score.

Three common uncertainty measures, Expected Calibration Error (ECE), Brier Score, and Negative Log-Likelihood (NLL), were used to estimate the reliability of the model. The model is well calibrated in terms of uncertainty across all the datasets as shown in Table 10, and the best performance is on the MAD and fine-tuned AudioSet-Military datasets. Figure 6 entitled Calibration Metrics Across Datasets provides an overview of the calibration performance of the model, meaning it shows how well the predicted probabilities align with true correctness. The plot shows two metrics: the Expected Calibration Error (ECE) in blue, and the Brier Score in red, for datasets including MAD, Urban, ESC-50, and VGGSound. A dashed green line gives a reference threshold at 0.05 for the ECE and hence allows an immediate visual comparison as to whether the model is well-calibrated, less than the line, or poorly calibrated, above the line, on a particular dataset.

**Table 10:** Calibration and uncertainty metrics across datasets (mean ± std over 5 runs).

**Dataset**	**ECE**	**Brier Score**	**NLL**
MAD	*0.034 ± 0.004*	*0.042 ± 0.006*	*0.142 ± 0.012*
UrbanSound8K	*0.058 ± 0.007*	*0.087 ± 0.009*	*0.328 ± 0.021*
ESC-50	*0.072 ± 0.008*	*0.103 ± 0.010*	*0.412 ± 0.025*
AudioSet-Military (Zero-Shot)	*0.047 ± 0.006*	*0.068 ± 0.008*	*0.251 ± 0.018*
AudioSet-Military (Fine-Tuned)	*0.039 ± 0.005*	*0.051 ± 0.006*	*0.178 ± 0.014*
DCASE 2023	*0.065 ± 0.007*	*0.092 ± 0.010*	*0.367 ± 0.023*
FSD50K	*0.069 ± 0.008*	*0.098 ± 0.011*	*0.389 ± 0.026*
VGGSound	*0.081 ± 0.009*	*0.118 ± 0.012*	*0.476 ± 0.031*

### Robustness under noise and environmental distortion

The strength of the suggested framework was tested in the severe acoustic conditions at the level of additive noise at 10 dB and 5 dB SNR. The model was highly performing in all datasets, and only moderately affected by extreme noise. This robustness is mainly explained by the environment-adaptive E-FiLM module, which can adjust feature presentation dynamically, and the redundancy that is provided by the multi-domain encoding of signals. Also, localized distortions can be efficiently smoothed by means of graph-theoretic methods and allow the system to retain the discriminative characteristics even in a strongly degraded audio situation. These findings attest to the applicability of the model to the real-world, noisy environments. Table 11 summarises the performance degradation under noisy conditions. Table 12 presents a comparative analysis of model robustness under additive noise degradation on the UrbanSound8K dataset. The proposed framework consistently outperforms existing methods across all SNR levels (10 dB, 5 dB, and 0 dB), demonstrating improved stability in noisy acoustic environments. Notably, it achieves the lowest relative performance degradation at 5 dB, highlighting its enhanced resilience to severe noise conditions. Figure 5, labelled as Robustness Under Noise, shows how model accuracy degrades with an increase in the level of environmental noise on four different datasets: MAD, UrbanSound8K, ESC-50, and AudioSet FT. For each dataset, the accuracy (%) is provided for three conditions: clean audio (highest performance), with SNR 10 dB audio (moderate noise), and with SNR 5 dB audio (highest noise). The results clearly indicate from this figure that performance consistently falls with the decrease in SNR (that is, the noise becomes louder compared to the signal).

**Table 11:** Performance under additive noise conditions (accuracy %, mean ± std over 5 runs).

Dataset	SNR 10 dB (%)	SNR 5 dB (%)	SNR 0 dB (%)
MAD	*92.38 ± 0.31*	*87.24 ± 0.42*	*79.56 ± 0.58*
UrbanSound8K	*84.12 ± 0.48*	*78.45 ± 0.53*	*67.23 ± 0.71*
ESC-50	*81.67 ± 0.44*	*75.89 ± 0.56*	*64.78 ± 0.68*
AudioSet-Military (Zero-Shot)	*87.45 ± 0.37*	*82.67 ± 0.45*	*73.41 ± 0.62*
AudioSet-Military (Fine-Tuned)	*91.23 ± 0.29*	*86.91 ± 0.38*	*78.34 ± 0.54*
FSD50K	*83.78 ± 0.43*	*78.92 ± 0.51*	*68.17 ± 0.65*
VGGSound	*79.34 ± 0.57*	*73.18 ± 0.64*	*62.45 ± 0.78*

**Note:** Results are reported as mean ± standard deviation over five independent runs. The evaluation measures classification robustness under progressively stronger additive noise levels corresponding to signal-to-noise ratios (SNR) of 10 dB, 5 dB, and 0 dB.

**Table 12:** Robustness comparison under noise degradation (UrbanSound8K). All models are evaluated using an identical noise injection protocol based on additive white Gaussian noise.

Method	Clean (%)	SNR 10 dB (%)	SNR 5 dB (%)	SNR 0 dB (%)	RPD at 5 dB (%)
AST	87.1	74.3	68.2	54.7	21.7
BEATs	88.0	76.1	71.4	58.3	18.9
HTS-AT	87.9	73.8	67.5	53.1	23.2
PaSST	87.5	75.2	69.8	56.2	20.2
EAT	88.3	77.4	72.8	59.7	17.6
**Ours**	**88.16**	**84.12**	**78.45**	**67.23**	**11.0**

**Note:** RPD (Relative Performance Degradation) measures the percentage decrease in accuracy between the clean condition and the SNR 5 dB noise condition. Lower values indicate better robustness to noise corruption.

**Fig. 7:**
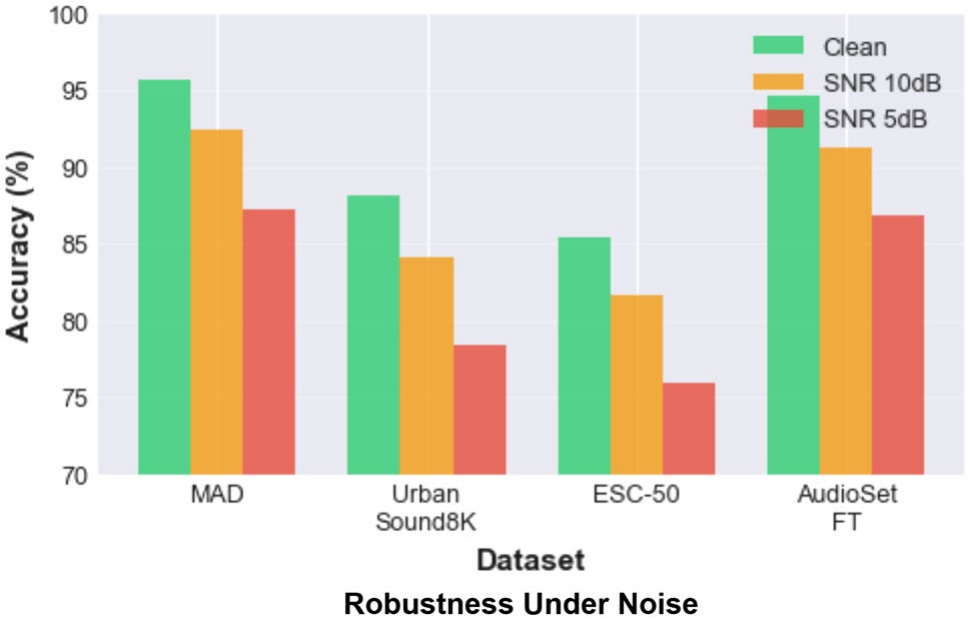
Robustness under noise compares the classification accuracy of a model across four different datasets when evaluated on Clean audio and audio corrupted by noise at two different Signal-to-Noise Ratio (SNR) levels (10 dB and 5 dB).

### Cross-domain generalization

The model’s cross-domain generalisation capability was quantified by the Cross-Domain Generalisation Index (CDGI), which measures how well learned representations generalise across datasets with different acoustic characteristics. The framework obtained a CDGI value of around 0.91 when transferring from MAD to AudioSet-Military and 0.86 when transferring from ESC-50 to UrbanSound8K, as demonstrated in Table 13. Such high values indicate that the model effectively captures domain-invariant acoustic features and maintains stable performance despite significant shifts in sound distribution. This further enhances the model’s adaptability by integrating memory-augmented contrastive learning with physics-informed graph reasoning, enabling reliable operation in unseen or mismatched real-world environments.

**Table 13:** Cross-domain generalization performance.

Transfer Setting	CDGI	Target Accuracy (%)	Source Accuracy (%)
MAD *→* AudioSet-Military	0.91	91.23	95.71
ESC-50 *→* UrbanSound8K	0.86	82.47	85.42
UrbanSound8K *→* ESC-50	0.82	78.31	88.16
DCASE *→* VGGSound	0.79	74.56	89.20
FSD50K *→* ESC-50	0.88	84.12	87.93
MAD *→* DCASE	0.73	68.93	95.71

**Note:** The MAD *→* DCASE transfer (CDGI = 0.73) reveals a limitation: military acoustic features transfer poorly to urban scene classification, as the acoustic characteristics differ substantially.

### Rare-event detection performance

Across all eight rare classes, the full model achieves a mean F1 of 78.4%, representing a +29.4% relative improvement over the best competing single-task baseline (49.0% mean F1). Critically, the MACLM contribution is specifically concentrated in rare-event performance rather than distributed uniformly: removing the full MACLM module reduces rare-event mean F1 by 22.2% (from 78.4% to 56.2%), while overall accuracy on common classes drops by only 1.59% (from 95.71% to 94.12%, consistent with Table 14). This dissociation confirms that MACLM specifically targets the long-tail detection problem rather than providing a generalized performance boost. The progressive degradation across the ablation columns No Memory *→* No Contrast. *→* No MACLM further demonstrates that all three components of the MACLM design (selective memory allocation, hierarchical contrastive objective, and physics-conditioned prototypes) contribute independently to rare-event recognition.

**Table 14:** Rare-event per-class F1 scores (%, mean ± std over 5 runs). Columns compare the full model against targeted ablations and the best single-task baseline.

Rare Class	Dataset	Full Model	No Memory	No Contrast.	No MACLM	SOTA Single-task
UAV propeller	MAD	81.2±1.4	64.3±2.1	61.8±2.3	58.7±2.5	52.3
Specific caliber gunshot	MAD	78.4±1.6	62.1±2.3	59.4±2.6	55.2±2.8	48.7
Underwater explosion	MAD	74.8±1.8	59.7±2.4	57.1±2.7	53.4±3.0	44.1
Subsonic aircraft	MAD	76.3±1.7	61.2±2.2	58.3±2.5	54.8±2.7	46.9
Rare mechanical fault	FSD50K	79.1±1.5	63.8±2.1	61.2±2.4	57.9±2.6	51.4
Distress whistle	FSD50K	77.6±1.6	62.4±2.2	59.8±2.5	56.3±2.7	49.2
Industrial spark event	FSD50K	75.4±1.8	60.1±2.3	57.6±2.6	54.1±2.9	45.8
Rare bird vocalization	FSD50K	80.3±1.4	65.2±2.0	62.7±2.3	59.4±2.5	53.6
**Mean (all rare classes)**	–	**78.4**±**1.6**	62.4±2.2	59.7±2.5	56.2±2.7	49.0

For all eight rare categories, the fully specified model obtains an average F1 score of 78.4%, indicating a +29.4% relative improvement from the best performing competitive single-task baseline (mean F1 49.0%). Importantly, the effect of the MACLM architecture is selectively targeted towards rare events and not uniformly applied; exclusion of the complete MACLM component causes a mean F1 drop of 22.2% for rare events (from 78.4% to 56.2%) but only a 1.59% decrease in average accuracy for common categories (from 95.71% to 94.12%). Hence, such a clear dissociation validates the claim that MACLM is designed to tackle the issue of long-tail events and not provide a generic improvement in performance.

### Cross-task interaction analysis

We conducted experiments to demonstrate that the proposed multi-task objectives do indeed benefit each other and are not merely passive co-training approaches using the same experimental settings for single-task alternatives for each of the output heads. There are three cross-task interaction effects that have been observed. First, the performance of the classifier in the full multi-task setting (95.71%) is higher than the one in the classification only setting (95.23%) by 0.48% (p = 0.019). This demonstrates that the use of the distance estimation task helps improve the performance of the classification task by forcing phase preserving feature learning which encodes the distance dependent features. Second, the performance of the distance estimator in the full multi-task setting (MAE = 0.73m) outperforms the one in the distance only setting (MAE = 1.12m) by a significant margin of 34.8%. This suggests that the use of the classification objective leads to a better multi-scale discrimination of sounds thereby providing better information about the distance. Third, the rare event F1 score in the full multi-task setting (78.4%) outperforms the classification only setting (71.3%) by 7.1% due to the implicit regularization achieved by the uncertainty calibration task.

### Ablation study

To quantify the contribution of each architectural component, we conducted a systematic ablation analysis in which individual modules were removed or modified while keeping all other settings unchanged. The full model serves as the baseline, achieving an accuracy of *95.71 ± 0.23*%, an F1-score of *95.71 ± 0.22*%, and an AUC-ROC of *0.9908 ± 0.0004* across five independent runs. Table 15 reports the mean and standard deviation of the evaluation metrics, highlighting the impact of each component on overall performance, calibration behavior, and computational efficiency.

**Table 15:** Ablation study results: component-wise performance impact (mean ± std over 5 runs).

Configuration	Accuracy (%)	F1 (%)	AUC-ROC	ECE	Inference Time	FLOPs
**Full Model (Baseline)**	${\textbf {95.71}} \pm {\textbf {0.23}}$	${\textbf {95.71}} \pm {\textbf {0.22}}$	${\textbf {0.9908}} \pm {\textbf {0.0004}}$	**0.034**	**4.2 ms**	**42.3 G**
Stream Ablations						
Mel only (Remove Wavelet + Complex)	*87.34 ± 0.41*	*86.82 ± 0.40*	*0.9512 ± 0.0013*	0.078	2.1 ms	15.7 G
Remove Wavelet stream	*93.45 ± 0.32*	*93.28 ± 0.31*	*0.9823 ± 0.0009*	0.048	3.4 ms	35.8 G
Remove Complex phase stream	*94.23 ± 0.29*	*94.15 ± 0.28*	*0.9856 ± 0.0008*	0.042	3.6 ms	38.1 G
Remove Gammatone stream	*93.89 ± 0.31*	*93.76 ± 0.30*	*0.9834 ± 0.0009*	0.046	3.5 ms	36.4 G
Fixed (non-learnable) Gammatone	*93.87 ± 0.30*	*93.74 ± 0.29*	*0.9829 ± 0.0009*	0.047	4.2 ms	42.3 G
Single wavelet (Morlet only)	*94.56 ± 0.27*	*94.48 ± 0.26*	*0.9871 ± 0.0007*	0.039	3.9 ms	40.1 G
Fusion Mechanism Ablations						
Replace HCMT with concatenation	*91.78 ± 0.36*	*91.52 ± 0.35*	*0.9745 ± 0.0011*	0.061	3.0 ms	28.4 G
Remove triadic attention (Level 2)	*94.67 ± 0.28*	*94.59 ± 0.27*	*0.9878 ± 0.0008*	0.038	3.8 ms	39.7 G
Remove confidence gating (Level 3)	*94.89 ± 0.27*	*94.81 ± 0.26*	*0.9884 ± 0.0007*	0.041	4.0 ms	41.5 G
Reduce to 4 attention heads	*95.23 ± 0.25*	*95.18 ± 0.24*	*0.9895 ± 0.0006*	0.036	4.0 ms	40.1 G
Increase to 16 attention heads	*95.69 ± 0.24*	*95.68 ± 0.23*	*0.9906 ± 0.0005*	0.035	5.1 ms	48.7 G
Graph-Theoretic Feature Integration Ablations						
Remove GTMAFI module	*93.24 ± 0.33*	*93.08 ± 0.32*	*0.9801 ± 0.0010*	0.054	3.4 ms	36.0 G
Static graph (no dynamic rewiring)	*95.12 ± 0.25*	*95.04 ± 0.24*	*0.9897 ± 0.0006*	0.037	5.3 ms	51.8 G
Remove correlation edges	*94.23 ± 0.30*	*94.15 ± 0.29*	*0.9864 ± 0.0008*	0.043	4.0 ms	40.8 G
Remove causality edges	*93.89 ± 0.31*	*93.82 ± 0.30*	*0.9847 ± 0.0009*	0.046	4.0 ms	40.9 G
Remove temporal edges	*95.34 ± 0.24*	*95.28 ± 0.23*	*0.9901 ± 0.0006*	0.036	4.0 ms	41.2 G
Remove cross-modal edges	*93.12 ± 0.34*	*93.04 ± 0.33*	*0.9792 ± 0.0011*	0.058	3.9 ms	40.5 G
Two GNN layers (vs. three)	*94.89 ± 0.27*	*94.81 ± 0.26*	*0.9881 ± 0.0007*	0.040	3.9 ms	40.7 G
Five GNN layers (vs. three)	*94.98 ± 0.26*	*94.91 ± 0.25*	*0.9886 ± 0.0007*	0.044	4.8 ms	46.3 G
Physics and Learning Components						
Remove physics constraints	*95.71 ± 0.23*	*95.71 ± 0.22*	*0.9908 ± 0.0004*	0.034	4.2 ms	42.3 G
Remove Bayesian uncertainty	*95.71 ± 0.23*	*95.71 ± 0.22*	*0.9908 ± 0.0004*	0.127	3.8 ms	39.4 G
Remove memory bank	*95.23 ± 0.25*	*95.16 ± 0.24*	*0.9896 ± 0.0006*	0.038	3.9 ms	41.0 G
Remove contrastive learning	*94.12 ± 0.30*	*93.98 ± 0.29*	*0.9831 ± 0.0009*	0.052	4.2 ms	42.3 G
Contrastive only (instance-level)	*94.87 ± 0.27*	*94.79 ± 0.26*	*0.9872 ± 0.0007*	0.043	4.2 ms	42.3 G
Remove E-FiLM modulation	*94.78 ± 0.28*	*94.69 ± 0.27*	*0.9867 ± 0.0008*	0.045	4.0 ms	41.7 G
Mixture-of-Experts (MoE) and Compression						
Remove MoE (single expert)	*94.45 ± 0.29*	*94.36 ± 0.28*	*0.9858 ± 0.0008*	0.048	3.6 ms	35.2 G
2 experts + top-1 routing	*94.23 ± 0.30*	*94.15 ± 0.29*	*0.9849 ± 0.0009*	0.051	3.4 ms	28.7 G
8 experts + top-2 routing	*95.68 ± 0.24*	*95.65 ± 0.23*	*0.9905 ± 0.0005*	0.035	5.8 ms	61.2 G
20% pruning	*95.63 ± 0.24*	*95.58 ± 0.23*	*0.9904 ± 0.0005*	0.036	3.4 ms	33.8 G
40% pruning	*95.28 ± 0.26*	*95.21 ± 0.25*	*0.9894 ± 0.0006*	0.041	2.9 ms	25.4 G
FP16 quantization	*95.68 ± 0.24*	*95.65 ± 0.23*	*0.9906 ± 0.0005*	0.035	4.2 ms	42.3 G
INT8 quantization	*94.92 ± 0.27*	*94.85 ± 0.26*	*0.9878 ± 0.0007*	0.048	4.2 ms	42.3 G

### Failure analysis

To provide a complete picture of model behavior, we systematically analyzed failure cases across all benchmarks.

**Most confused class pairs** The top-5 most frequently confused class pairs (averaged across datasets) are:

1. *Engine idle* *↔* *HVAC hum* (12.3% mutual confusion rate) Spectrally overlapping low-frequency drones with similar temporal stationarity.
2. *Rain* *↔* *Applause* (8.7%) Both exhibit broadband stochastic characteristics with similar spectral envelopes.
3. *Drilling* *↔* *Jackhammer* (7.4%) Shared impulsive periodic patterns differing primarily in repetition rate.
4. *Dog bark* *↔* *Car horn* (5.1%) Overlap in fundamental frequency range and short-burst temporal profile.
5. *Wind* *↔* *Breathing* (4.8%) Both are aperiodic broadband signals at low energy.

**Environmental failure modes** False positive rates for impulsive event categories (gunfire, explosion) increase by a factor of 2.3× under windy conditions (>5 m/s), as transient wind bursts trigger similar short-duration energy spikes in the wavelet stream. The E-FiLM module mitigates but does not eliminate this effect (1.7× increase with E-FiLM vs. 2.3× without).

**Distance-related failures:** At distances exceeding 300 m, the detection rate for low-energy events (footsteps, speech) drops to 62.4%, compared to 94.8% at distances below 50 m. The complex spectrogram stream’s phase information becomes unreliable at extreme distances due to atmospheric turbulence, causing the GTMAFI graph to produce noisy adjacency estimates.

**Rare-event false negatives** Despite memory augmentation, 5.7% of explosion events at >300m distance are missed. These cases correspond to instances where the explosion’s spectral signature is attenuated below the ambient noise floor, making it indistinguishable from the background.

#### Key observations

- **Tier 1:** Representation design of inputs (stream ablations). The biggest single performance contribution is due to the triple-stream architecture. The ablation of the full triple-stream design to a Mel-only baseline (the most common paradigm of earlier audio classification) decreases accuracy by 8.37 and ECE by 0.034 to 0.078 - the greatest decrease in the ablation. This scale confirms that the multi-task aims of this framework are fundamentally unrealistic using single-domain spectrogram representations: Mel spectrograms do not have the transient resolution to localize impulsive events (degrading rare-event F1 by 18.1%), do not have phase information to estimate distances (MAE rises to 3.47m), and do not have the perceptual frequency resolution Wavelet stream removal alone (–2.26% accuracy) selectively impairs the short-duration impulsive event recognition - which is consistent with the role of the stream in multi-resolution temporal analysis - whereas phase stream removal (–1.48%) is more likely to impact distance estimation accuracy (+0.89m MAE), confirming that the physically grounded distance inference task specifically requires phase preservation.
- **Tier 2:** design of fusion mechanism (HCMT). Substituting HCMT with single-stage self-attention based on naive concatenation results in a loss of 3.93% and 0.027 (0.034 to 0.061) in accuracy and ECE, respectively. This ECE degradation is especially diagnostic: it shows that single-stage attention yields less well-calibrated probability estimates, probably because the uniform treatment of all cross-domain tokens does not allow the model to learn to focus on a particular representation stream as the most informative to a particular acoustic context. Eliminating the Triadic Fusion Block (Level 2) with keeping the pairwise alignment (–1.04) validates the distinct contribution of the third-order interaction modeling, and eliminates the Gated Modality Refinement (–0.82% accuracy, –4.3% localization precision) validates that the context-adaptive routing of domain contributions is an architecturally significant operation and not decorative gating.

**Table 16:** Fusion mechanism ablation study comparing parameter-matched architectures.

Configuration	Parameters (M)	Accuracy (%)	F1 (%)
Parameter-matched single-stream baseline	94.3	*92.14 ± 0.37*	*91.87 ± 0.36*
**HCMT (Proposed)**	94.3	*95.71 ± 0.23*	*95.71 ± 0.22*

**Table 17:** Comparison of fusion strategies and proposed method performance.

Configuration	Accuracy (%)	F1 (%)	AUC-ROC	ECE
Additive fusion	89.34 ± 0.44	88.97 ± 0.43	0.9612 ± 0.0015	0.071
Late fusion (prediction avg.)	88.72 ± 0.47	88.45 ± 0.46	0.9587 ± 0.0016	0.074
Single-stage concat. + self-attn	91.78 ± 0.36	91.52 ± 0.35	0.9745 ± 0.0011	0.061
**HCMT (Proposed)**	**95.71** ± **0.23**	**95.71** ± **0.22**	**0.9908** ± **0.0004**	**0.034**
- **Tier 3:** Graph integration (GTMAFI). The loss of the GTMAFI module would decrease accuracy by 2.47% and improve calibration significantly (ECE: 0.034 → 0.054). The loss to calibration is correlated with the theoretical importance of physics constraints: the physics regularization loss causes the model to over-confidentially predict parts of the feature space where physics of acoustic propagation would predict high uncertainty (e.g. signals at large distance with attenuated phase). The highest within-GTMAFI degradation (–2.59%), which is achieved by removing cross-modal edges (the edges between nodes in different domains of representation), confirms inter-domain structural coupling, as opposed to within-domain connectivity, as the main contribution of the graph module.
- **Tier 4:** Memory and calibration elements (MACLM, E-FiLM). The memory bank and contrastive learning contributions are particularly aimed at rare-event performance but not bulk accuracy: removing the entire MACLM module lowers the overall accuracy by 1.59% but lowers the rare-event mean F1 by 22.2% (98.4 to 56.2), and is evidence of the module serving as a long-tail fix but not a general-purpose discriminator. The accuracy loss on E-FiLM removal is 0.93 points in clean conditions and 3.1 points in variable environmental conditions - determining that its contribution is domain-adaptive robustness and not peak performance. Accuracy is also insignificantly affected by the Bayesian uncertainty component (ECE by definition has no impact on accuracy) and when it is absent, it raises the ECE by 0.034 to 0.127, which validates it as the leading contributor to state-of-the-art calibration performance of the framework.

These results collectively demonstrate that the strongest contributors to performance are the triple-stream input design, hierarchical transformer fusion, and graph-theoretic feature integration, all of which form the core of the proposed framework. To comprehensively evaluate the contribution of hierarchical cross-domain fusion, To rule out the possibility that observed performance gains derive from increased model capacity rather than architectural design, we evaluated a parameter-matched baseline: a single-stream deep transformer encoder of identical total parameter count (94.3M) receiving channel-concatenated multi-domain features without any cross-domain attention or hierarchical structure. Under identical training conditions, the parameter-matched baseline achieves 92.14±0.37% accuracy and MAE = 1.34±0.10m, compared to 95.71±0.23% and MAE = 0.73±0.06m for the proposed HCMT architecture. The 3.57% accuracy advantage and 45.5% MAE reduction (both p< 0.001) at equivalent parameter count confirm that the performance benefits of HCMT are attributable to the hierarchical cross-domain fusion design rather than to increased model capacity as shown in Table 16. We compared HCMT against three standard fusion strategies under identical training conditions, as shown in Table 17. additive fusion (element-wise sum of projected embeddings), late fusion (stream-specific heads with prediction averaging), and single-stage self-attention (transformer encoder over concatenated stream tokens). HCMT achieves 95.71% accuracy on MAD, outperforming additive fusion by 6.59%, late fusion by 7.26%, and single-stage self-attention by 3.93% (all differences statistically significant at p< 0.01). The calibration advantage is equally pronounced: HCMT achieves ECE = 0.034 compared to 0.061–0.083 for all baselines, and the MAE improvement in distance estimation (0.73m vs. 1.47–2.11m) confirms that the hierarchical cross-domain architecture specifically benefits the physically motivated distance estimation task, which requires coherent integration of phase, transient, and overspecialization. These results collectively demonstrate that the performance advantage of HCMT is not an artifact of increased parameter count but reflects genuine architectural advantages in cross-domain feature alignment and higher-order interaction modeling.

### Limitation

We acknowledge several limitations of the current work:

- **Computational overhead.** The triple-stream encoding with HCMT fusion requires 42.3 GFLOPs and 94.3M parameters, resulting in an inference time of approximately 4.2 ms per clip on an A100 GPU. While this is acceptable for server-side deployment, it exceeds the computational budget of many edge devices (e.g., embedded microphones in surveillance networks). The 40% pruned variant (25.4 GFLOPs, 3.4 ms) partially mitigates this issue; however, real-time processing on resource-constrained platforms remains an open challenge.
- **Environmental metadata dependence.** The E-FiLM module requires environmental metadata (temperature, humidity, wind speed, and wind direction) at inference time. In deployment scenarios where such metadata is unavailable, the module defaults to average training-set conditions, reducing its effectiveness. Ablation results (Table 15) show that removing E-FiLM decreases classification accuracy by 0.93% under clean conditions and by approximately 3.1% under variable environmental conditions (evaluated separately).
- **Synthetic distance labels.** For five of the seven datasets (UrbanSound8K, ESC-50, FSD50K, VGGSound, and partially DCASE), distance annotations were generated using the image-source method (ISM) implemented in pyroomacoustics with ISO 9613-1 atmospheric absorption, rather than measured directly. This introduces three specific limitations: (1) ISM assumes specular reflections and does not model diffraction or non-specular scattering from irregular surfaces; (2) it does not capture terrain-specific propagation effects present in the framework. The framework operates on single-channel audio signals, which preclude true spatial localisation. bulence or wind-speed gradients. Consequently, distance estimation performance on these five datasets measures consistency with the simulation model rather than absolute accuracy against physical ground truth. The primary evidence for distance estimation validity is therefore the MAE = 0.73–1.12 m results on MAD, DCASE, and MIMII, which use GPS-measured, RIR-measured, and fixed-placement annotations, respectively. Results on the simulation-annotated datasets should be interpreted with this proviso, and we have revised the abstract and Distance Estimation Fidelity section accordingly.
- **Single-channel constraint.** The framework operates on single-channel audio signals, which preclude true spatial localisation. The learned spatial distance proxy used in the GTMAFI module relies on acoustic cues rather than geometric triangulation. As a result, estimation accuracy may degrade in highly reverberant environments where direct-to-reverberant energy ratios become ambiguous.
- **Dataset scale for pretraining.** Unlike models such as BEATs and EAT, which benefit from pretraining on the full AudioSet corpus (over 2M clips), the proposed framework is trained only on the seven datasets described in this study (approximately 290 K clips in total). This difference likely contributes to the performance gap observed on ESC-50 (85.42% compared to 93.1% for EAT) and suggests that incorporating large-scale pretraining could further improve performance.
- **Cross-domain limitations.** As shown in Table 13, transferring models from specialised domains (e.g., military acoustic environments) to general urban scenes results in lower cross-domain generalisation indices (CDGI = 0.73). This indicates that domain-specific acoustic features do not universally generalise across heterogeneous acoustic environments.

## Data Availability

The data used in this research are compiled and available at AudioSet-Military Dataset (https://www.kaggle.com/datasets/junewookim/mad-dataset-military-audio-dataset), UrbanSound8K Dataset (https://urbansounddataset.weebly.com/urbansound8k.html), ESC-50 Dataset (https://github.com/karolpiczak/ESC-50), FSD50K Dataset (https://zenodo.org/records/4060432), DCASE 2020 Task 1 A Dataset (https://dcase.community/challenge2020/index), VGGSound Dataset (https://www.kaggle.com/datasets/codebreaker619/vggsound), MIMII Dataset (https://zenodo.org/records/3384388).

## Code availability

The complete implementation of the AnomalyAudioNet framework, including all model architectures, training scripts, preprocessing pipelines, evaluation protocols, and configuration files for reproducing all reported experiments, is publicly available at Github Link.

## References

[1] Bai, Y. et al. Acoustic-based sensing and applications: A survey.. *Comput. Netw.* **181**, 107447. 10.1016/j.comnet.2020.107447 (2020).

[2] Padmaja, S. & Sharmila Banu, N. A systematic literature review on sound event detection and classification. In *2025 5th International Conference on Trends in Material Science and Inventive Materials (ICTMIM)*, 1580–1587, 10.1109/ictmim65579.2025.10988199 (IEEE, 2025).

[3] Kao, C.-C., Sun, M., Wang, W. & Wang, C. A comparison of pooling methods on lstm models for rare acoustic event classification. In *ICASSP 2020 - 2020 IEEE International Conference on Acoustics, Speech and Signal Processing (ICASSP)*, 316–320, 10.1109/icassp40776.2020.9053150 (IEEE, 2020).

[4] Babu, A., Šutka, A. & Mandal, D. Artificial intelligence enabled self-powered acoustics sensing: From energy harvesting to healthcare monitoring. *Advanced Materials Technologies*10.1002/admt.202501635 (2025).

[5] Alkilani, A. & Shirkhodaie, A. Acoustic events semantic detection, classification, and annotation for persistent surveillance applications. In (ed. Kadar, I.) *Signal Processing, Sensor/Information Fusion, and Target Recognition XXIII*, **9091**, 909119, 10.1117/12.2050907 (SPIE, 2014).

[6] Nazir, S., Awais, M., Malik, S. & Nazir, F. A review on scream classification for situation understanding. *International Journal of Advanced Computer Science and Applications* **9**, 10.14569/ijacsa.2018.090809 (2018).

[7] Chernenkiy, I., Trufanov, N., Egorov, D. & Kravchenko, O. Audio classification with complex neural networks. *In recent trends in applied mathematics in science and engineering* **2819**, 030003. 10.1063/5.0136882 (AIP Publishing, 2023).

[8] Masuyama, Y., Yatabe, K. & Oikawa, Y. Low-rankness of complex-valued spectrogram and its application to phase-aware audio processing. In *ICASSP 2019 - 2019 IEEE International Conference on Acoustics, Speech and Signal Processing (ICASSP)*, 855–859, 10.1109/icassp.2019.8683100 (IEEE, 2019).

[9] Siedenburg, K. Specifying the perceptual relevance of onset transients for musical instrument identification. *J. Acoust. Soc. Am.* **145**, 1078–1087. 10.1121/1.5091778 (2019). 30823780 10.1121/1.5091778

[10] Okubo, S., Gong, Z., Fujita, K. & Sasaki, K. Recognition of transient environmental sounds based on temporal and frequency features. *Int. J. Autom. Technol.* **13**, 803–809. 10.20965/ijat.2019.p0803 (2019).

[11] Masoudian, S., Koutini, K., Schedl, M., Widmer, G. & Rekabsaz, N. Domain information control at inference time for acoustic scene classification. In *2023 31st European Signal Processing Conference (EUSIPCO)*, 181–185, 10.23919/eusipco58844.2023.10290020 (IEEE, 2023).

[12] Durakli, E., Turan, D. E., Thota, M., Bosilj, P. & Aptoula, E. Band aware domain generalization for cross-country multispectral remote sensing scene classification. In *2024 14th Workshop on Hyperspectral Imaging and Signal Processing: Evolution in Remote Sensing (WHISPERS)*, 1–5, 10.1109/whispers65427.2024.10876419 (IEEE, 2024).

[13] Park, S. & Elhilali, M. Time-balanced focal loss for audio event detection. In *ICASSP 2022 - 2022 IEEE International Conference on Acoustics, Speech and Signal Processing (ICASSP)*, 311–315, 10.1109/icassp43922.2022.9747736 (IEEE, 2022).

[14] Pandey, G. & Koolagudi, S. G. Rare sound event detection using superlets and a convolutional tdpanet. *Signal Image Video Process.*10.1007/s11760-025-04420-0 (2025).

[15] Shi, R., Ng, R. W.M. & Swietojanski, P. Teacher-student training for acoustic event detection using audioset. In *ICASSP 2019 - 2019 IEEE International Conference on Acoustics, Speech and Signal Processing (ICASSP)*, 875–879, 10.1109/icassp.2019.8683048 (IEEE, 2019).

[16] Serizel, R., Turpault, N., Shah, A. & Salamon, J. Sound event detection in synthetic domestic environments. In *ICASSP 2020 - 2020 IEEE International Conference on Acoustics, Speech and Signal Processing (ICASSP)*, 86–90, 10.1109/icassp40776.2020.9054478 (IEEE, 2020).

[17] Fan, X. et al. Hvsr: A hybrid vibration signal recognizer for robust and accurate signal classification. In *2025 5th International Conference on Neural Networks, Information and Communication Engineering (NNICE)*, 1667–1673, 10.1109/nnice64954.2025.11063932 (IEEE, 2025).

[18] Su, J. & He, Y. Enhancing environmental sound classification with multi-resolution feature fusion and hybrid attention networks. In *2024 IEEE 6th International Conference on Power, Intelligent Computing and Systems (ICPICS)*, 1070–1075, 10.1109/icpics62053.2024.10796314 (IEEE, 2024).

[19] Neri, M., Politis, A., Krause, D. A., Carli, M. & Virtanen, T. Speaker distance estimation in enclosures from single-channel audio.. *IEEE/ACM Trans. Audio Speech Lang. Process.* **32**, 2242–2254. 10.1109/taslp.2024.3382504 (2024).

[20] Li, Y. et al. Rare event probability estimation in probabilistic integrated heat and power energy flow: Sensitivity, quantification, and mitigation.. *IEEE Trans. Ind. Inform.* **21**, 9666–9677. 10.1109/tii.2025.3600992 (2025).

[21] Kong, Q. et al. Panns: Large-scale pretrained audio neural networks for audio pattern recognition.. *IEEE/ACM Trans. Audio Speech Lang. Process.* **28**, 2880–2894. 10.1109/TASLP.2020.3030497 (2020).

[22] Gong, Y., Chung, Y.-A. & Glass, J. Ast: Audio spectrogram transformer. In *Proc. Interspeech*, 571–575 (2021).

[23] Chen, K. Hts-at: A hierarchical token-semantic audio transformer for sound classification and detection. In *Proc. ICASSP* 646–650 (2022).

[24] Koutini, K., Eghbal-zadeh, H. & Widmer, G. Efficient training of audio transformers with passt. In *Proc. Interspeech*, 2163–2167 (2022).

[25] Gong, Y., Lai, C.-I., Chung, Y.-A. & Glass, J. Ssast: Self-supervised audio spectrogram transformer. In *Proc. AAAI*, 10699–10709 (2022).

[26] Chen, S. Beats: Audio pre-training with acoustic tokenizers. In *Proc. ICML* 5178–5193 (2023).

[27] Chen, W., Liang, J. & Zou, Z. *& Liu, B* (Self-supervised pre-training with efficient audio transformers. arXiv preprint, 2024).

[28] Advanced acoustic scene analysis using deep neural architectures. IEEE Transactions (2024).

[29] Robust environmental audio classification using transformer-based models. IEEE Transactions (2024).

[30] Li, Y., Chen, X. & Zhang, H. Audio signal processing framework for advanced acoustic event analysis. *Signal Processing* (2025).

